# Synthesis of novel 1,2,4-thiadiazinane 1,1-dioxides *via* three component SuFEx type reaction†

**DOI:** 10.1039/c8ra07627h

**Published:** 2018-11-07

**Authors:** Mzilikazi F. Khumalo, Ekemini D. Akpan, Praveen K. Chinthakindi, Edikarlos M. Brasil, Kamal K. Rajbongshi, Maya M. Makatini, Thavendran Govender, Hendrik G. Kruger, Tricia Naicker, Per I. Arvidsson

**Affiliations:** Catalysis and Peptide Research Unit, University of KwaZulu Natal Durban South Africa 4001 Naickert1@ukzn.ac.za; Molecular Sciences Institute, School of Chemistry, University of the Witwatersrand PO Witz 2050 Johannesburg South Africa; Science for Life Laboratory, Drug Discovery & Development Platform & Division of Translational Medicine and Chemical Biology, Department of Medical Biochemistry and Biophysics, Karolinska Institutet Stockholm Sweden per.arvidsson@scilifelab.se

## Abstract

Herein, we report the preparation of 1,2,4-thiadiazinane 1,1-dioxides from reaction of β-aminoethane sulfonamides with dichloromethane, dibromomethane and formaldehyde as methylene donors. The β-aminoethane sulfonamides were obtained through sequential Michael addition of amines to α,β-unsaturated ethenesulfonyl fluorides followed by further DBU mediated sulfur(vi) fluoride exchange (SuFEx) reaction with amines at the S–F bond.

## Abstract 2

Herein, we report the preparation of 1,2,4-thiadiazinane 1,1-dioxides from reaction of β-aminoethane sulfonamides with dichloromethane, dibromomethane and formaldehyde as methylene donors.

## Graphical abstract

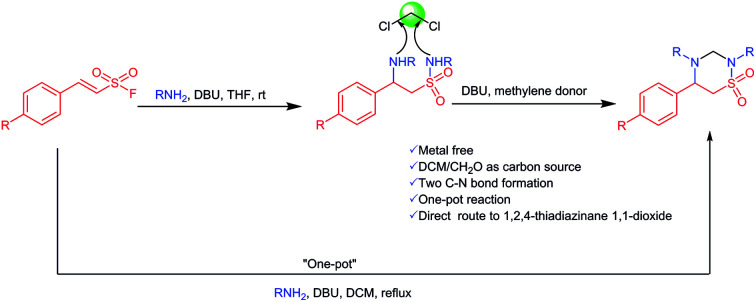

The 1,2,4-thiadiazinane 1,1-dioxide motif can be found in many biologically active compounds for vastly different medical conditions. For example, verubecestat (1) has been in phase III clinical trials as a β-amyloid precursor protein cleaving enzyme (BACE 1) inhibitor to treat moderate and prodromal Alzheimer's disease.^1^ Ribizzi *et al.* have shown that taurolidine (2) displays cytotoxic activity against certain human tumour cells,^2^ but primarily it is used as an antibacterial agent.^3^ In addition, benzothiadiazines (3) are patented as ATP-sensitive potassium channel modulators for the treatment of respiratory, central nervous, and endocrine system disorders.^4^ 1,2,4-Thiadiazinane 1,1-dioxides of this type may be formed by various methods;^5–13^ most closely related to the present work is the [2 + 2 + 2] sulfa Staudinger cycloaddition of sulfonylchlorides and imines, in which case β-sultams may also be formed through the corresponding [2 + 2] cycloaddition.^14,15^ α,β-Unsaturated sulfonyl fluorides 4 are so far rarely encountered as starting materials for organic synthesis.^16–18^ The literature on this reagent describe it as a connector molecule,^19^ and a warhead in chemical biology.^20–22^ There are only four publications that, so far, have reported the use of α,β-unsaturated sulfonyl fluoride based compounds as starting materials in organic synthesis.^23–26^ Based on our earlier experience with the reactivity of aryl α,β-unsaturated sulfonyl fluoride towards various amine nucleophiles^17^ (Scheme 1), we hypothesized that an α,β-unsaturated sulfonyl fluoride of type 4 can possibly be explored for the synthesis of thiadiazinanes. This hypothesis was based on observation of low amounts of the six-membered product was formed along with the major β-sultam product 5 when *p*-nitrophenylethenesulfonyl fluoride was subjected to excess methyl amine in methylene chloride as a solvent and triethylamine as additional base at room temperature (Scheme 1).
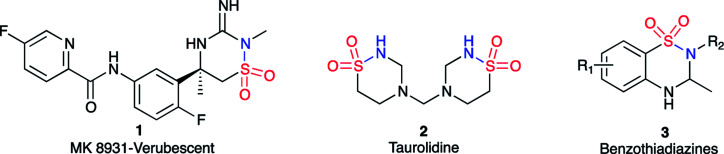


**Scheme 1:**
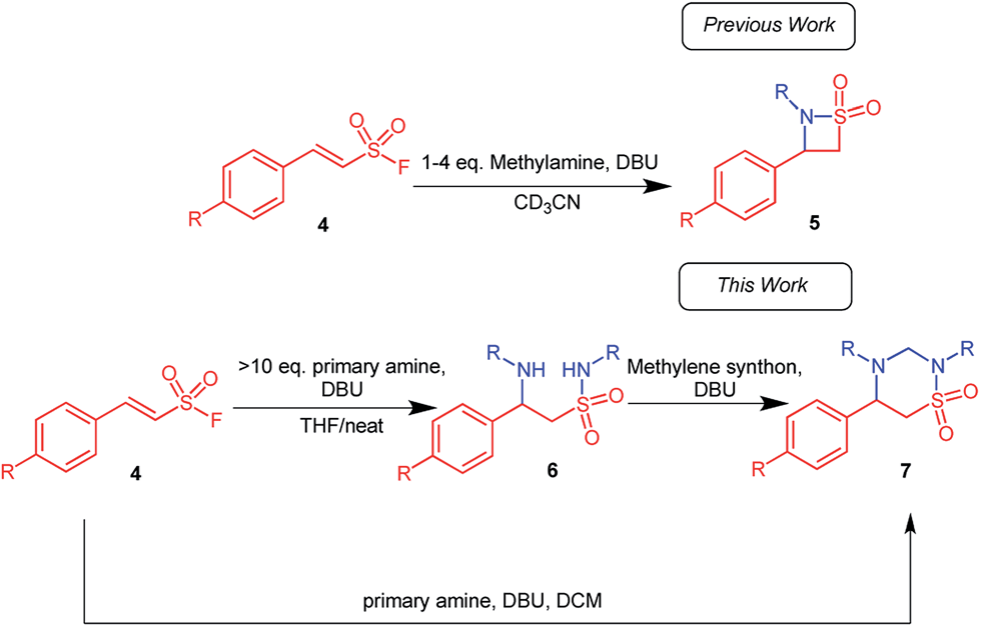
Formation of 1,2,4-thiadiazinane 1,1-dioxides, along with β-sultams, when aryl ethenesulfonyl fluorides are subjected to large excess of primary amines in DCM as solvent and DBU as catalyst.

The reactivity of dichloromethane (DCM) as a methylene donor was unfamiliar to us at the time, but a literature survey quickly revealed that organic solvents (DMF,^27^ DMSO,^28–30^ CHCl_3_ (ref. 31 and 32) and CH_2_Cl_2_ (ref. 33 and 34)) have proved to be more than solvents. DCM has indeed been reported to act as a *bis*-electrophilic methylene donor in the presence of strong bases and nucleophiles^33^ (*e.g.* carboxylic acids,^35^ thiols,^36^ amines, *etc.*). DCM may also form hydrochloride salts,^37^ aminals,^38^ and quaternary salts^39^ when reacted with tertiary and secondary amines. These reactions were reviewed by Mills *et al.*^40^ and the kinetics of the reaction of DCM with pyridine was documented by Rudine *et al.*^41^ Liu and co-workers reported formation of methylene-bridged 3,3′-bis-(oxazolidin-2-one) through reaction of oxazolidin-2-ones with DCM and sodium hydride.^42^ Cui *et al.* reported the synthesis of bispidine with the utilisation of DCM as a C1 unit.^43^ Dipyrrolidylmethane CH_2_(pyr)_2_ and dipiperidylmethane, CH_2_(pip)_2_ were synthesized *via* the condensation of the secondary amine precursors and DCM at room temperature in the absence of light.^44^ Another reaction of amines with methylene chloride yielded aminals rapidly.^45^ Matsumoto *et al.* reported the reaction of DCM with ketones or esters in the presence of secondary amines at high pressure whereby DCM was used as methylene bridge in forming both C–C and C–N bonds.^46^ Zhang and co-workers also published the formation of simultaneous carbon–carbon bond and carbon-nitrogen bonds whereby DCM acted as a synthon in the presence of 1,8-diazabicyclo [5.4.0] undec-7-ene (DBU) and a copper catalyst.^47^

## Results and discussion

Intrigued by the observed 1,2,4-thiadiazinane 1,1-dioxide formation under certain reaction conditions we decided to investigate the scope of this novel transformation. Herein, we report the optimized synthesis of 1,2,4-thiadiazinane 1,1-dioxides (7) *via* (*para*)-aryl substituted ethene sulfonyl fluorides (ESF) with different amines using DCM, formaldehyde, or DBM as the methylene bridge sources and DBU as the catalyst.

**Scheme 2:**
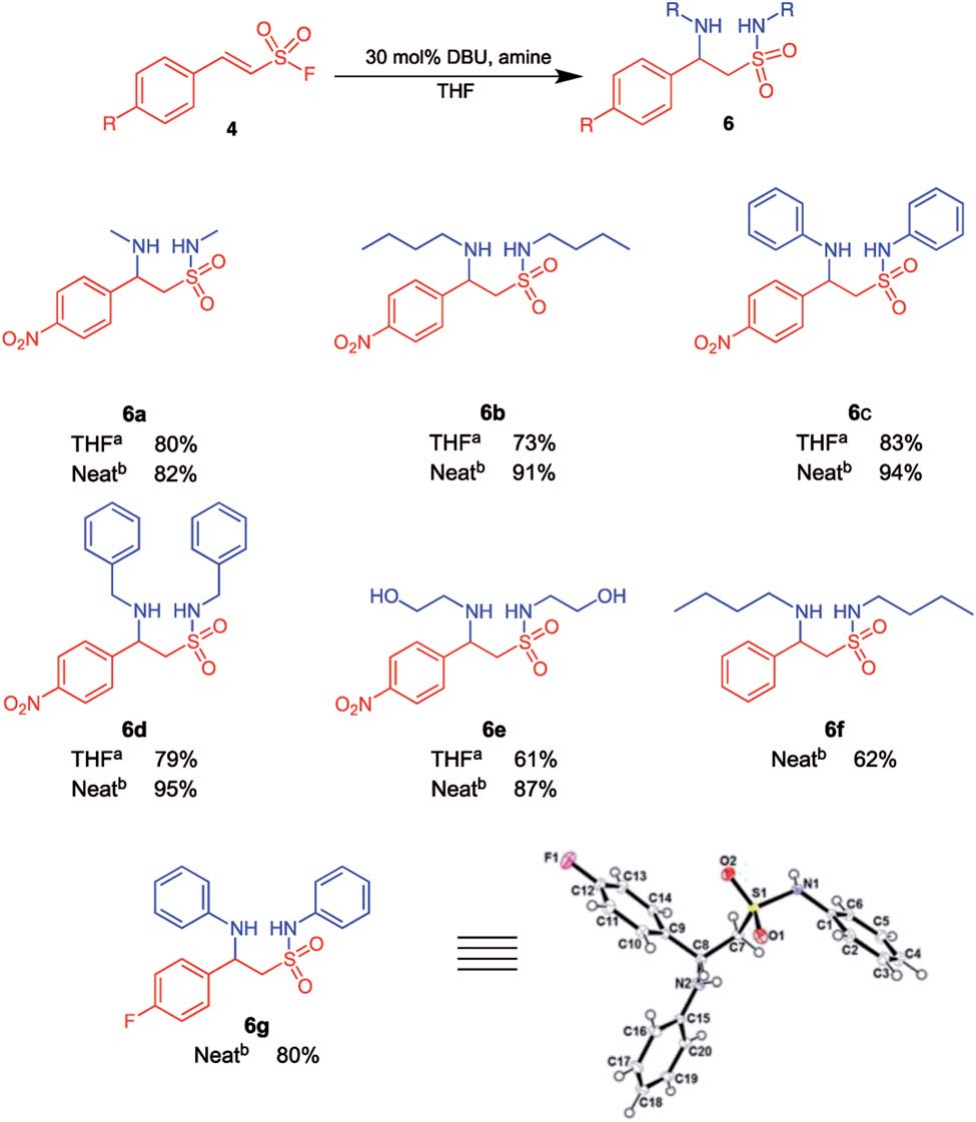
β-Aminoethane sulfonamides may be isolated when the reaction if carried out with THF or neat amine as solvent. ^*a*^10 Equiv. amine, 30 mol% DBU, 5 ml THF, overnight, room temperature; ^*b*^neat reaction conditions, 10 equiv. amine, 30 mol% DBU, 3–5 h, room temperature.

For the optimization, it was important to understand the reaction sequence involved. Consequently, we first performed a series of experiments to establish that the reaction proceeds *via* the expected β-aminoethane sulfonamide intermediate 6 (Scheme 1): It was observed that when 10 equiv. of the amine as well as 15 mol% DBU were used, a more polar product formed in small quantity. This product was identified by liquid chromatography mass spectrometry (LC/MS) and confirmed by NMR analysis to be the β-aminoethane sulfonamide intermediate 6. Therefore, in an effort to isolate the β-aminoethane sulfonamide intermediate, methylene chloride was replaced with tetrahydrofuran (THF) as solvent with 30 mol% DBU. The reaction successfully gave the β-aminoethane sulfonamide intermediate in good yields (61–83%) along with traces of the β-sultam at room temperature (Scheme 2).

A neat reaction that involved *para*-nitrophenylethenesulfonyl fluoride 4 and an amine in the presence of 30 mol% DBU also successfully yielded the β-aminoethane sulfonamide intermediate in excellent yields (62–95%) that is also depicted in Scheme 2.

We also attempted to ascertain the effect of substituents on the phenyl ring of 4 on the formation of β-aminoethane sulfonamide 6. Compound 6f was isolated in 62% yield from the reaction of (*E*)-2-phenylethenesulfonyl fluoride with butyl amine (10 equiv.) in the presence of 30 mol% DBU (Scheme 2). This drop in yield could be attributed to the absence of the electron withdrawing nitro substituent, resulting in a less efficient Michael addition and sulfonamide formation. Substrates with electron donating substituents on the aryl ring gave little to no product formation under these conditions; this is in accordance with studies by Qin *et al.*, who concluded that the Michael reaction of secondary amines on β-arylethenesulfonyl fluorides required EWG on the aryl group when performed in THF or DCM.^26^ Further, the electron withdrawing fluoro substituent on the phenyl ring of 4 was evaluated and compound 6g was isolated in 80% yield using aniline as the amine of choice. All structures of compounds appearing in Scheme 2 was unequivocally deduced from full spectroscopic characterization. In addition, we also obtained crystals of product 6g suitable for single crystal X-ray diffraction.^48^ Interestingly, the fluorophenyl and phenylamino groups appeared to be attached almost perpendicular to each other with the torsion angle (C1–N1–S1–C7) of −70.12 (11)°.

**Scheme 3:**
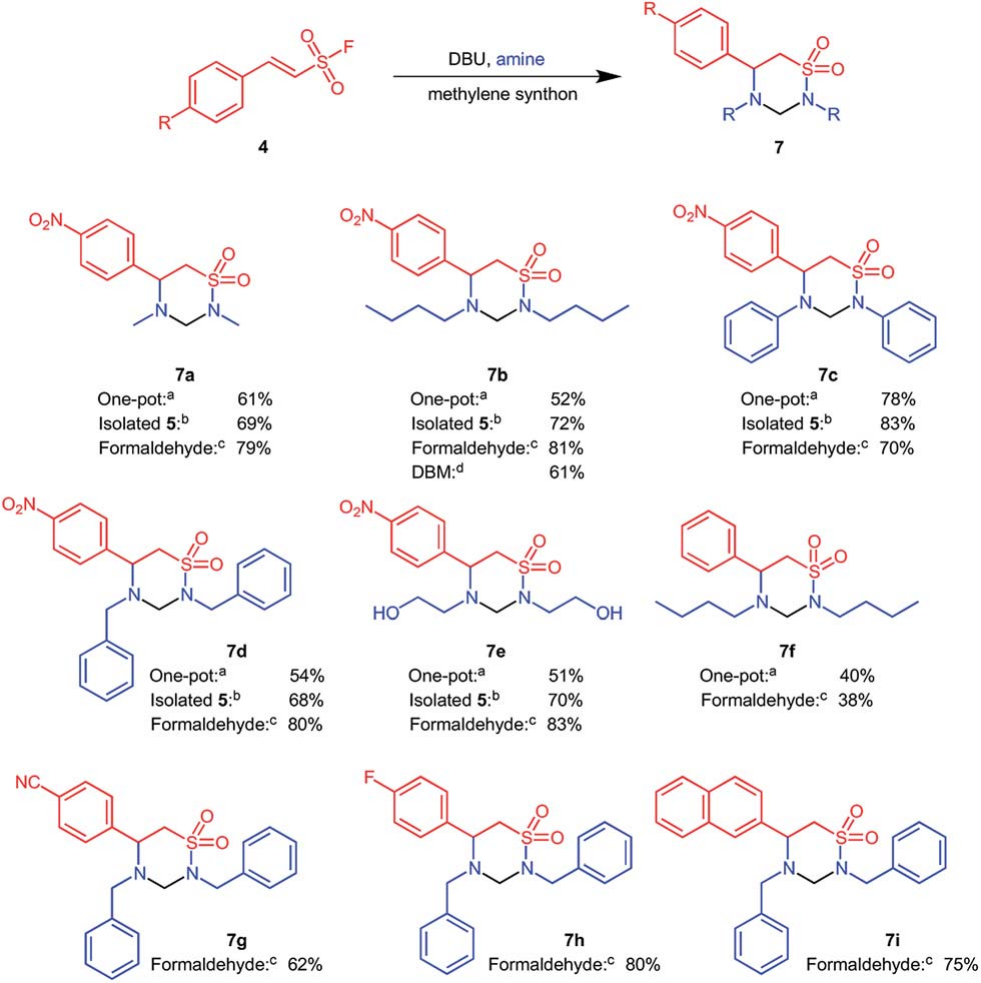
Formation of 1,2,4-thiadiazinane 1,1-dioxides under one-pot conditions in DCM or using a two-step procedure with other methylene donors for cyclization.^*a*^(One pot) 10 equiv. amine, 50 mol% DBU, 5.0 ml DCM, reflux; ^*b*^β-aminoethane sulfonamide, 20 mol% DBU, 5 ml DCM, reflux; ^*c*^β-aminoethane sulfonamide, cat. acetic acid, 1.1 equiv. CH_2_O, 3 ml EtOH or MeOH microwave 90 °C, 200 W, 5 min; ^*d*^β-aminoethane sulfonamide, 20 mol% DBU, 5 ml DBM, reflux. Note for b–d, the yield refers to the final step.

Having understood the reactions steps involved, we next varied the base in the one pot reaction to obtain the 1,2,4-thiadiazinane 1,1-dioxides 7 according to Scheme 3.

The best conversion was obtained with 50 mol% DBU, whereas 15 and 100 mol% DBU gave comparable conversion values. DBU appeared to be less effective at 100 mol% towards the formation of 1,2,4-thiadiazinane 1,1-dioxide 7. Chen *et al.* recently showed, with the aid of deuterium labelling experiments, that an excess amount of DBU favours the loss of the sulfonyl group *via* a benzylic shift.^25^ This might be associated with the low conversion to the six membered cyclic product observed with stoichiometric amounts of DBU, however the mechanism of the reaction reported herein is not yet fully established.

In the case of 15 mol% DBU, it seemed that the amount of DBU was inadequate for a complete cyclization of the β-amino ethanesulfonamide 6 intermediate, as evidenced from the presence of 6 observed from TLC and *via* mass spectral analysis of the reaction mixture even after 24 and 48 hours. A range of bases were then screened at 50 mol% but none gave convincing conversions. For example, pyridine gave a conversion of 30 : 70 four membered : six membered products after five days of reaction time; hence DBU remained our choice of base. The temperature was varied while keeping 10 equiv. butyl amine and 50 mol% DBU. An 80% conversion towards the thiadiazinane 7 was obtained at reflux in DCM. The best conditions obtained after optimization was 50 mol% DBU; 10 equiv. butyl amine; 24 hours; under reflux in DCM. Increasing the amount of amines above 10 equiv. gave a lower yield; this is probably due to formation of a too harsh reaction mixture that cause loss of sulfonyl group as reported by Chan *et al.*^25^

Under the one pot conditions, aniline as a nucleophile achieved a 100% conversion towards the six membered 1,2,4-thiadiazinane 1,1-dioxides 7c, and the product could be isolated in 78% yield. However, methylamine (7a), butyl amine (7c), benzyl amine (7d), and 2-aminoethanol (7e) nucleophiles all gave a mixture of the four membered β-sultam ring and the desired six membered 1,2,4-thiadiazinane 1,1-dioxides (Scheme 3-conditions a). Under this one-pot procedure, (*E*)-2-phenylethenesulfonyl fluoride and butyl amine resulting in a modest 40% yield of 7f, reflecting a combination of a worse Michael reaction with this substrate (*cf.*Scheme 2) and the β-sultam formation with *n*-butylamine. Despite optimization, the one-pot transformation of ESF derivatives to 1,2,4-thiadiazinane 1,1-dioxides continued to yield a mixture of the four membered β-sultam 5 and the desired 6-membered product 7 for all aliphatic amines investigated. We therefore decided to revert to a two-step procedure, in which the intermediate β-aminoethane sulfonamide 6 was isolated before cyclization with DCM as a methylene donor. As mentioned earlier, when DCM was replaced with THF, or ran neat at room temperature, 6 was obtained in optimum quantity. Preceding, 6 was cyclized with DCM whereby the first attempt was conducted at room temperature but yielded only <10% of 7. Thereafter, the cyclization of 6 was carried out using 20 mol% catalytic DBU in DCM under reflux, and offered 1,2,4-thiadiazinane 1,1-dioxides 7 in 68–83% yield within 8–12 h (Scheme 3-conditions b). The two-step procedure proved only marginally more efficient overall than the one-pot procedure, *i.e.* product 7a was obtained in 56% overall yield *vs.* 61% in one-pot, product 7b 65% (overall) *vs.* 52% (one-pot), 7c 78% (overall) *vs.* 78% (one-pot), 7d 65% (overall) *vs.* 54% (one-pot), 7e 61% (overall) *vs.* 51% (one-pot), and 7f 24% (overall) *vs.* 40% (one-pot). Despite not offering much improved overall yield, the two step procedure simplified purification as a minute amount of the 4-membered β-sultam was formed under these conditions; however, the operational simplicity of the one-pot could make it the preferential protocol.

Thereafter, other methylene donors were evaluated for cyclization of the β-aminoethane sulfonamide 6 to 1,2,4-thiadiazinane 1,1-dioxides. Formaldehyde, in the presence of acetic acid under microwave conditions, successfully cyclized β-aminoethane sulfonamide derivatives 6 to the corresponding 1,2,4-thiadiazinane 1,1-dioxides in 38–83% yield (Scheme 3-conditions c). The ability of dibromomethane (DBM) and chloroform to cyclize 6 to 7 were also compared in relation to DCM. 1,2,4-Thiadiazinane 1,1-dioxide 6b reacted with dibromomethane in the presence of 50 mol% DBU at reflux to give 7b 61% yield (Scheme 3-conditions d). In contrast, reacting 6b under similar conditions in chloroform gave only trace amounts of the corresponding 1,2,4-thiadiazinane 1,1-dioxide after two days as identified by LC/MS. In an attempt to form the 2-oxy derivative of 7, the β-aminoethane sulfonamide 6 was reacted with 1,1-carbonyldiimidazole (CDI) for cyclization; the main product in this case was the activated amine which did not undergo intramolecular cyclization with the sulfonamide functionality.

Overall, we conclude that the best conditions for formation of the desired 1,2,4-thiadiazinane 1,1-dioxides was through isolation of the intermediate 6 followed by ring-closure to 7 with formaldehyde as a methylene donor. We utilized this optimized protocol for the conversion of the two electron withdrawing ESF derivatives 6g and 6h, as well as the naphthyl analogue 6i, to yield the corresponding 1,2,4-thiadiazinane 1,1-dioxides 7g–7i (Scheme 3).

## Conclusions

We have explored rarely utilized α,β-unsaturated ethenesulfonyl fluorides to synthesize novel β-aminoethane sulfonamides and developed a convenient procedure for the synthesis of 1,2,4-thiadiazinane 1,1-dioxides in moderate to good yields. Although we initially identified the 6-membered product as a rare example of DCM reactivity as methylene donor, the optimized protocol make use of formaldehyde as one carbon source. We anticipate that the reported methods will aid the synthesis of thiadiazinane derivatives that will find applications as biologically active compounds.

## Conflicts of interest

There are no conflicts to declare.

## Supplementary Material

RA-008-C8RA07627H-s001

RA-008-C8RA07627H-s002
