# Supplementary material for: Synthesis of novel 1,2,4-thiadiazinane 1,1-dioxides via three component SuFEx type reaction
Source: RSC Adv. 2018 Nov 7;8(65):37503–7. doi: 10.1039/c8ra07627h (PMC9089429; doi:10.1039/c8ra07627h)
Supplement: RA-008-C8RA07627H-s001 [file RA-008-C8RA07627H-s001.pdf]

Electronic Supplementary Material (ESI) for RSC Advances.
This journal is © The Royal Society of Chemistry 2018

Synthesis of novel 1, 2, 4-thiadiazinane 1, 1-dioxides via three component SuFEx type reaction

Mzilikazi F. Khumalo[a], Ekemini D. Akpan[a], Praveen K. Chinthakindi[a], Edikarlos M. Brasil[a], Kamal K. Rajbongshi[a], Maya M. Makatini[b], Thavendran Govender[a], Hendrik G. Kruger[a], Tricia Naicker*[a], Per I. Arvidsson*[a,c]

[a]Catalysis and Peptide Research Unit, University of KwaZulu-Natal, Durban, South Africa
[b]Molecular Sciences Institute, School of Chemistry, University of the Witwatersrand, P.O Box Wits 2050, Johannesburg, South Africa
[c]Science for Life Laboratory, Drug Discovery & Development Platform & Division of Translational Medicine and Chemical Biology, Department of Medical Biochemistry and Biophysics, Karolinska Institutet, Stockholm, Sweden

Supporting Information

1 General Information: 2
1.1 Typical procedure for the synthesis of (*E*)-2-(4-nitrophenyl)ethenesulfonyl fluoride (4) 2
1.1.1 Method I: 2
1.1.2 Method II: 2
1.2 Typical procedure for the synthesis of *β*-amionethane sulfonamide intermediates (6a–6g) 3
1.2.1 Method I: 3
1.2.2 Method II: 3
1.3 General procedure for the synthesis of 1,2,4–thiadiazinane 1,1–dioxides (7a–7i) 3
1.3.1 Method I: 3
1.3.2 Method II: 3
1.3.3 Method III: 3
1.4 Characterization 5
1.5 References 11
Spectral data .... **Error! Bookmark not defined.**

1 General Information:

All commercially available starting materials (Pd-catalysts, ethenesulfonyl fluoride, butyl amine, benzyl amine, methylamine, aniline, 1,8-Diazabicyclo [5.4.0] undec-7-ene (DBU), and aryl boronic acids) were purchased from various suppliers. Methylamine solution (40 wt. %/Water) was extracted into DCM and the rest of the chemicals were used as purchased. Where applicable, phosphorus pentoxide and sodium/benzophenone was used to dry DCM and THF respectively. All chemical reactions were monitored by LC/MS and TLC (aluminum sheet coated with silica gel 60 F_{254}, visible at 254 nm under ultra-violet light. A simple gravity column chromatography utilizing silica gel 60 mesh as a stationary phase and ethyl acetate in hexane as eluting solvents were set for purification. ^{1}H, ^{13}C NMR spectra were recorded on Bruker Advance III 400 MHz instrument in $CDCl_3$ using the residual signals from $CDCl_3$ as the reference. LCMS-HRMS was recorded using on Bruker microTOF-Q II instrument operating at ambient temperatures by using a sample concentration of approximately 1.0 ppm.

.

1.1 Typical procedure for the synthesis of (*E*)-2-(4-nitrophenyl)ethenesulfonyl fluoride (4)

1.1.1 Method I:

A sealed tube under a nitrogen atmosphere was charged with ethenesulfonyl fluoride (121.7 µL, 1.47 mmol, 2.5 equiv), $Pd(OAc)_2$ (82 mg, 0.1 mol, 0.2 equiv), $Cu(OAc)_2$ (178 mg, 0.98 mmol, 2 equiv), LiOAc (38 mg, 0.59 mmol, 1.2 equiv), and 2.0 mL of dry THF. Subsequently, 4-nitro boronic acid (82 mg, 0.49 mmol, 1 equiv.) was dissolved in dry THF (2.0 mL), and added to the reaction mixture drop wise using a syringe over ~30 min. The reaction mixture was stirred at room temperature for 3-5 hours and the reaction progress was monitored using TLC. Upon completion, the reaction mixture was filtered through a plug of Celite. The filtrate was concentrated under vacuum and purified through silica gel 60 mesh column chromatography using 2-4% ethyl acetate in hexane as eluent to obtain the pure (*E*)-2-(4-nitrophenyl)ethenesulfonyl fluoride.

1.1.2 Method II:

Synthesis was carried out using a modified literature procedure.[1] An oven dried 20 mL round bottom flask (RBF) charged with AcOH (5 mL), 4-nitro boronic acid (50 mg, 0.31 mmol, 1 equiv,), $AgNO_3$ (102 mg, 0.60 mmol, 2 equiv), $Pd(OAc)_2$ (3.36 mg, 0.05 mol, 6 equiv), and ethenesulfonyl fluoride (6.0 equiv) was fitted with a condenser under atmospheric conditions and allowed to reflux at 80 °C overnight. Upon the complete consumption of the 4-nitro boronic acid, brine (5-10 mL) was added and the (*E*)-2-(4-nitrophenyl)ethenesulfonyl fluoride precipitate filtered under vacuum and washed with water to achieve the pure product as a light yellow solid.

1.2 Typical procedure for the synthesis of *β*-amionethane sulfonamide intermediates (6a–6g)

1.2.1 Method I:

A 20 mL RBF was charged with (*E*)-2-(4-nitrophenyl) ethenesulfonyl fluoride in anhydrous THF (5 mL). DBU (30 mol%) and the respective amine (10 equiv.) were added. The reaction was allowed to proceed for overnight at room temperature and thereafter washed with water and extracted three times with 10 mL diethyl ether. Solvent was removed in vacuum to obtain the crude product. The crude product was purified by column chromatography with a gradient of 5–20% ethyl acetate/hexane.

1.2.2 Method II:

Respective amine (10 equiv) was added to a 10 mL RBF charged with (*E*)-2-(4-nitrophenyl) ethenesulfonyl fluoride (0.09 mmol), 30 mol% DBU and the reaction was allowed to run for an hour at room temperature. The reaction progress was monitored with TLC, and upon completion washed with water and extracted three times with 10 mL of diethyl ether. Solvent was removed under vacuum to obtain the crude product. The crude product was purified by column chromatography with a gradient of 5–35% ethyl acetate/hexane.

1.3 General procedure for the synthesis of 1,2,4–thiadiazinane 1,1–dioxides (7a–7i)

1.3.1 Method I:

A 10 mL RBF was charged with (*E*)-2-(4-nitrophenyl) ethenesulfonyl fluoride (20 mg, 0.09 mmol), DBU (50 mol%), the respective amine (10 equiv) and 5 mL of dry DCM. The reaction mixture was stirred at 50 °C for 24 hours. The reaction progress was monitored with TLC, and upon completion was washed with water and extracted three times with 10 mL of ethyl acetate. The crude product was purified on the neutral alumina column using 5–25% ethyl acetate in hexane as an eluent to obtain the pure 1,2,4–thiadiazinane 1,1–dioxides.

1.3.2 Method II:

A 10 mL RBF was charged with the isolated ethanesulfonamide intermediate, DBU (20 mol%) and DCM (5 mL), and the reaction mixture was stirred at 50 °C for 8–12 hours. The reaction progress was monitored with TLC, and upon completion was washed with water and extracted three times with 10 mL of ethyl acetate. The crude product was purified on the neutral alumina column using 5–25% ethyl acetate in hexane as an eluent to obtain the pure 1,2,4–thiadiazinane 1,1–dioxides.

1.3.3 Method III:

A mixture of the isolated ethanesulfonamide intermediate, catalytic amount of acetic acid, formaldehyde and EtOH or MeOH (3 mL) was subjected to microwave heating (90 °C, 200 watts) for 5 mins. The reaction progress was monitored with TLC. Upon completion, washed with water and extracted three times with 10 mL of ethyl acetate. The crude product was purified on a neutral

alumina column using 5–25% ethyl acetate in hexane as an eluent to obtain the pure 1,2,4–thiadiazinane 1,1–dioxides.

Table 1. Optimization of the reaction conditions.[a]

ENTRY	SOLVENT	EQUIV. AMINE	BASE (mol%)	TEMP. (°C)	RATIO[b] (7:5)
	**Reaction time: Two days**	**$BuNH_2$**	**DBU**		
**1**	DCM	3	15	r.t	71:29
**2**	DCM	10	15	r.t	41:59
**3**	DCM	20	15	r.t	66:34
**4**	DCM	10	50	r.t	27:73
**5**	DCM	10	100	r.t	42:58
**6**	DCM	10	DABCO[d]	r.t	34:66
**7**	DCM	10	TEA[e]	r.t	62:38
**8**	DCM	10	Pyridine[f]	r.t	35:65
	**Reaction time: Five days[c]**				
**9**	DCM	10	DABCO[d]	r.t	33:67
**10**	DCM	10	TEA[e]	r.t	61:39
**11**	DCM	10	Pyridine[f]	r.t	30:70
	One day		DBU		
**12**	DCM	10	50	0 °C	40:60
**13 BEST CONDTIONS**	**DCM**	**10**	**50**	**reflux**	**20:80**

[a]Reaction conditions: amine, base, temperature, 5 mL DCM, [b]Conversion ratio based on ^{1}H NMR analysis of the crude reaction mixture (**7** and **5**); [c]the same reaction analysed after two days was allowed to remain stirring for five days prior to ^{1}H NMR analysis, [d,c,f]50 mol**%** used.

1.4 Characterization

(*E*)-2-(4-nitrophenyl)ethenesulfonyl fluoride (4)

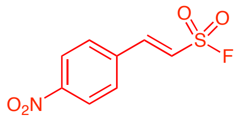

The ^{1}H, ^{13}C and ^{19}F NMR are identical to those reported in the previous paper.[2]

N-methyl-2-(methylamino)-2-(4-nitrophenyl)ethanesulfonamide (6a)

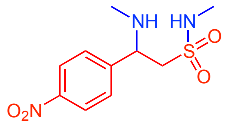

According to the general procedure (**1.2.2**), method II, (*E*)-2-(4-nitrophenyl)ethenesulfonyl fluoride (20.0 mg, 0.09 mmol) treated with methylamine (10 equiv) and 30 mol% DBU (0.027 mmol), for 1–3 h yielded compound **6a** (19.0 mg, 82%) as a yellow solid, R_f = 0.36 (20% ethyl acetate/hexane), **m.p**. 128–129 °C. δ 8.23 (2H, d, *J* = 8.0 Hz), 7.55 (2H, d, *J* = 8.1 Hz), 4.25 (1H, d, *J* = 9.8Hz), 3.33-3.29 (m,1H), 3.13 (d, 1H, J = 14.3Hz), 2.80 (3H, s), 2.25 (3H, s) ppm. **^{13}C APT NMR ($CDCl_3$, 100 MHz):** δ 148.4, 147.7, 128.0 (d), 124.2 (d), 60.1, 57.1, 34.2, 29.3. **HRMS (ESI):** m/z calcd for $C_{10}H_{15}N_3O_4S$ $[M+H]^+$ 274.0862, found 274.0856.

N-butyl-2-(butylamino)-2-(4-nitrophenyl)ethanesulfonamide (6b)

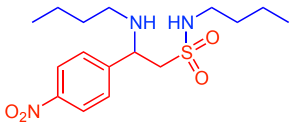

According to the general procedure (**1.2.2**), method II, (*E*)-2-(4-nitrophenyl)ethenesulfonyl fluoride (15.0 mg, 0.065 mmol) treated with butyl amine (10 equiv) and 30 mol% DBU (0.0195 mmol), for 1–3 h yielded compound **6b** (17.0 mg, 91%) as a dark brown solid, R_f = 0.37 (20% ethyl acetate/hexane), **m.p**. 115–117 °C. **^{1}H-NMR ($CDCl_3$, 400 MHz):** δ 8.23 (2H, d, *J* = 8.7 Hz), 7.56 (2H, d, *J* = 8.7 Hz), 4.46 (1H, b), 4.36 (1H, dd, *J* = 4.3 Hz), 3.29 (1H, m), 3.11 (3H, m), 2.46 (1H, m), 2.34 (1H, m), 1. 52 (2H, m), 1.35 (7H, m), 0.93 (3H, t), 0.87 (3H, t) ppm. **^{13}C APT NMR ($CDCl_3$, 100 MHz):** δ 149.2, 147.7, 128.1(d), 124.3 (d), 58.5, 58.4, 47.2, 43.1, 32.2 (d), 20.1 (d), 13.7 (d), ppm. **HRMS (ESI):** m/z calcd for $C_{16}H_{27}N_3O_4S$ $[M+H]^+$ 358.1801, found 358.1795.

2-(4-nitrophenyl)-*N*-phenyl-2-(phenylamino)ethanesulfonamide (6c)

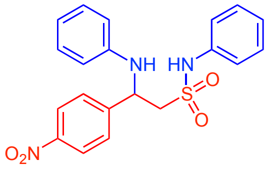

According to the general procedure (**1.2.2**), method II, (*E*)-2-(4-nitrophenyl)ethenesulfonyl fluoride (20.0 mg, 0.09 mmol) treated with aniline (10 equiv) and 30 mol% DBU (0.027 mmol), for 1–3 h yielded compound **6c** (31.2 mg, 94%) as a brown solid, R_f = 0.35 (20% ethyl acetate/hexane), **m.p.** 136–137 °C. **^{1}H NMR ($CDCl_3$, 400 MHz):** δ 8.17 (2H, d, *J* = 8.6 Hz), 7.49 (2H, d, *J* = 8.6 Hz), 7.36 (2H, t, *J* = 7.6 Hz), 7.22 (1H, t, *J* = 7.6 Hz), 7.14 (4H, m), 6.77 (1H, t, *J* = 7.6 Hz), 6.49 (2H, d, *J* = 7.6 Hz), 5.03 (1H, t, *J* = 4.4 Hz), 4.82 (1H, s), 3.48 (2H, m) ppm. **^{13}C APT NMR ($CDCl_3$, 100 MHz):** δ 148.1, 145.6, 130.1, 129.6 (d) 127.3 (d), 126.0 (d), 124.6 (d), 120.7(d), 119.5, 114.4 (d), 57.0, 54.6. **HRMS (ESI):** m/z calcd for $C_{20}H_{19}N_3O_4S$ $[M+H]^+$ 398.1175, found 398.1169.

***N*-benzyl-2-(benzylamino)-2-(4-nitrophenyl)ethanesulfonamide (6d)**

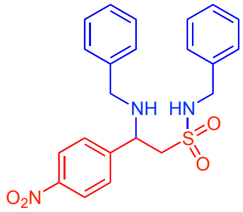

According to the general procedure (**1.2.2**), method II, (*E*)-2-(4-nitrophenyl)ethenesulfonyl fluoride (20.0 mg, 0.09 mmol) treated with benzyl amine (10 equiv) and 30 mol% DBU (0.027 mmol), for 1–3 h yielded compound **6d** (29.3 mg, 95%) as a brown oil, R_f = 0.61 (15% ethyl acetate/hexane). **^{1}H-NMR ($CDCl_3$, 400 MHz):** δ 8.19 (2H, d, *J* = 8.6 Hz), 7.52 (1H, m), 7.33 (6H, m), 7.21 (5H, q), 4.48 (1H, m), 4.34 (1H, m), 4.18 (1H, m) 3.63 (1H, d, *J* = 13.2 Hz), 3.43 (1H, d, *J* = 13.2 Hz), 3.22 (1H, q), 2.99 (1H, dd, *J* = 15.0 Hz) ppm. **^{13}C APT NMR ($CDCl_3$, 100 MHz):** δ 148.5, 147.8, 140.1, 139.1, 129.0 (d), 128.6 (d), 128.3 (d), 128.2 (d), 127.9 (d), 127.5, 124.2 (d), 59.1, 57.3, 51.2, 47.4 ppm. **HRMS (ESI):** m/z calcd for $C_{22}H_{23}N_3O_4S$ $[M+H]^+$ 426.1488, found 426.1482.

***N*-(2-hydroxyethyl)-2-(2-hydroxyethylamino)-2-(4-nitrophenyl)ethanesulfonamide (6e)**

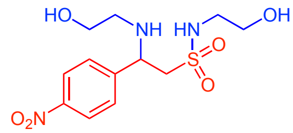

According to the general procedure (**1.2.1**), method I, (*E*)-2-(4-nitrophenyl)ethenesulfonyl fluoride (20.0 mg, 0.09 mmol) treated with ethanol amine (10 equiv) and 30 mol% DBU (0.027 mmol), THF (5 mL) for 24 h gave the compound **6e** (17.6 mg, 61%) as a gold oil, R_f = 0.31 (60% ethyl acetate/hexane). **^{1}H-NMR (MeOD, 400 MHz):** δ 8.24 (2H, d, *J* = 8.4 Hz), 7.68 (2H, d, *J* = 8.3 Hz), 4.40-4.37 (1H, m), 3.64-3.57 (4H, m), 3.53-3.47 (2H, m), 3.39-3.34 (1H, m), 3.21-3.17 (2H, m), 2.62-2.56 (1H, m), 2.51-2.45 (1H, m), **^{13}C APT NMR (MeOD, 100 MHz):** δ ppm. 150.4, 149.0, 129.7 (d), 124.8 (d), 62.2, 61.8, 59.6, 58.8, 50.1, 46.3 ppm. **HRMS:** m/z calcd for $C_{12}H_{19}N_3O_6S$ $[M+H]^+$ 334.1073, found 334.1067.

***N*-butyl-2-(butylamino)-2-phenylethanesulfonamide (6f).**

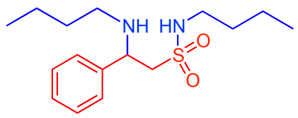

According to the general procedure (**1.2.2**), method II, (*E*)-2-phenylethenesulfonyl fluoride (40.0 mg, 0.21 mmol) treated with butyl amine (10 equiv) and 30 mol% DBU (0.063 mmol), for 1–3 h gave the compound **6f** (41.7 mg, 62%) as a light brown oil, R_f = 0.34 (15% ethyl acetate/hexane), **^{1}H-NMR ($CDCl_3$, 400 MHz):** δ 7.39 (5H, m), 4.21 (1H, q, *J* = 4.4 Hz), 3.33 (1H, m), 3.17 (1H, dd, *J* = 4.3 Hz), 3.01 (2H, m), 2.44 (2H, ddd), 1.37 (8H, m), 0.88 (6H, m) ppm. **^{13}C APT NMR ($CDCl_3$, 100 MHz):** δ 141.8, 129.0 (d), 128.1, 126.8 (d), 65.9, 58.9, 57.8, 47.0, 42.9, 32.1, 20.3, 20.2, 13.6 (d), ppm. **HRMS (ESI):** m/z calcd for $C_{16}H_{28}N_3O_4S$ $[M+H]^+$ 313.1701, found 313.1695.

2-(4-fluorophenyl)-N-phenyl-2-(phenylamino)ethanesulfonamide (6g)

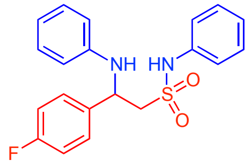

According to the general procedure (**1.2.2**), method II, (*E*)-2-(4-flourophenyl)ethenesulfonyl fluoride (20.0 mg, 0.09 mmol) treated with aniline (10 equiv) and 30 mol% DBU (0.027 mmol), for 1–3 h gave the compound **6g** (26.64 mg, 80%) as brown solid, R_f = 0.52 (30% ethyl acetate/hexane), **m.p.** 127–128 °C.**^{1}H-NMR ($CDCl_3$, 400 MHz):** δ 7.34 (2H, t, *J* = 7.83 Hz), 7.28-7.25 (3H, m), 7.19 (1H, t, *J* = 7.45 Hz), 7.12-7.09 (3H, m), 7.00 (2H, t, *J* = 8.56Hz), 6.75 (1H, t, *J* = 7.40 Hz), 6.54 (2H, d, *J* = 7.88 Hz), 6.37 (1H, b), 4.94 (1H, dd, *J* = 3.55), 4.65 (1H, b), 3.53-3.41 (2H, m) ppm. **^{13}C APT NMR ($CDCl_3$, 100 MHz):** δ ppm. 146.1, 136.4, 129.9 (d), 129.5 (d), 128.0, 125.6 (d), 120.5 (d), 119.2 (d), 116.3, 114.4(d), 57.3, 54.4 ppm. **HRMS (ESI):** m/z calcd for $C_{20}H_{19}N_2O_2S$ $[M+H]^+$ 371.1230, found 371.1224

2,4-dimethyl-5-(4-nitrophenyl)-1,2,4-thiadiazinane 1,1-dioxide (7a)

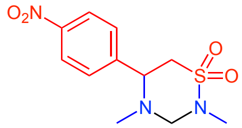

According to the general procedure (**1.3.1**), method I, (*E*)-2-(4-nitrophenyl)ethenesulfonyl fluoride (15.0 mg, 0.05 mmol) treated with methylamine (10 equiv), DBU (50 mol%) in 5 mL of dry DCM at 50 °C for 24 hours gave the compound **7a** (9.6 mg, 61%) as white solid, R_f = 0.33 (25% ethyl acetate/hexane), **m.p.** 109–110 °C. **^{1}H-NMR ($CDCl_3$, 400 MHz):** δ 8.25 (2H, d, *J*= 7.93 Hz), 7.54 (2H, d, *J* = 8.0 Hz), 4.33 (1H, d, *J* = 12.7 Hz), 3.92-3.90 (2H, m), 3.17-3.05 (5H, m), 2.01 (3H, s) ppm. **^{13}C APT NMR ($CDCl_3$, 100 MHz):** δ ppm 147.9, 146.6, 128.1 (d), 124.5 (d), 73.7, 65.8, 50.1, 38.5, 36.5. **HRMS (ESI):** m/z calcd for $C_{11}H_{15}N_3O_4S$ $[M+H]^+$ 286.0862, found 286.0877.

2,4-dibutyl-5-(4-nitrophenyl)-1,2,4-thiadiazinane 1,1-dioxide (7b)

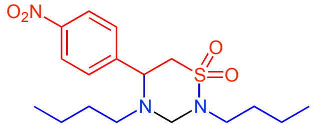

According to the general procedure (**1.3.2**), method II, *N*-butyl-2-(butylamino)-2-(4-nitrophenyl)ethanesulfonamide (15.0 mg, 0.04 mmol) treated with DBU (20 mol%) in 5 mL of dried DCM at 50 °C

for 24 hours gave the compound **7b** (8.1 mg, 72%) as brown solid, R_f = 0.32 (25% ethyl acetate/hexane), **m.p**. 111–112 °C. **^{1}H-NMR (CDCl$_3$, 400 MHz):** δ 8.22 (2H, d, *J* = 8.6 Hz), 7. 52 (2H, d, *J* = 8.7 Hz), 4.25 (1H, d, *J* = 12.9 Hz), 4.16-4.07(2H, m), 3.38-3.28 (2H, m), 3.11-3.02 (2H, m), 2.35-2.30 (1H, m), 2.09-2.02 (1H, m), 1.65-1.55 (2H, m), 1.46-1.28 (4H, m), 1.19-1.03 (2H, m), 0.95 (3H, t, *J* = 7.3 Hz), 0.76 (3H, t, *J*= 7.63Hz) ppm. **^{13}C APT NMR (CDCl$_3$, 100 MHz):** δ 147.8, 147.0, 128.3 (d), 124.4 (d), 67.4, 64.3, 52.1, 48.9, 47.6, 30.8, 28.5, 20.1, 19.8, 13.8, 13.6 ppm. **HRMS (ESI):** m/z calcd for $C_{17}H_{27}N_3O_4S$ $[M+H]^+$ 370.1801, found 370.1795.

5-(4-nitrophenyl)-2,4-diphenyl-1,2,4-thiadiazinane 1,1-dioxide (7c)

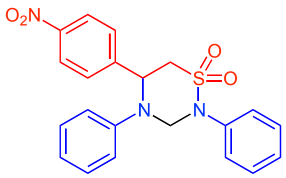

According to the general procedure (**1.3.2**), method I, 2-(4-nitrophenyl)-*N*-phenyl-2-(phenylamino)ethanesulfonamide (20.0 mg, 0.05 mmol) treated with DBU (20 mol%) in 5 mL of dried DCM at 50 °C for 8-12 hours gave the compound **7c** (12.6 mg, 83%) as grey solid, R_f = 0.30 (25% ethyl acetate/hexane), **m.p**. 139–140 °C. **^{1}H-NMR (CDCl$_3$, 400 MHz):** δ 8.12 (2H, d, *J* = 8.0 Hz), 7.86 (2H, d, *J* = 8.0 Hz), 7.54 (2H, d, *J* = 7.8 Hz), 7.48 (2H, t, *J* = 7.8 Hz), 7.38 (1H, t, *J* = 7.8 Hz), 7.21 (2H, t, *J* = 7.5 Hz), 7.12 (2H, d, *J* = 7.5 Hz), 7.05 (1H, t, *J* = 7.5 Hz), 5.23- 5.16 (2H, m), 4.79 (1H, d, *J* = 13.3), 3.45-3.35 (2H, m) ppm. **^{13}C APT NMR (CDCl$_3$, 100 MHz):** δ 147.5, 146.2, 146.0, 141.0, 129.6 (d), 129.3(d), 128.4(d), 127.8, 126.3(d), 125.9, 124.6(d), 124.2(d), 73.6, 62.7, 52.9, ppm. **HRMS (ESI):** m/z calcd for $C_{21}H_{19}N_3O_4S$ $[M+H]^+$ 410.1175, found 410.1169.

2,4-dibenzyl-5-(4-nitrophenyl)-1,2,4-thiadiazinane 1,1-dioxide (7d)

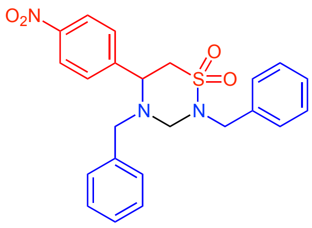

According to the general procedure (**1.3.3**), method III, *N*-benzyl-2-(benzylamino)-2- (4-nitrophenyl)ethanesulfonamide (20.0 mg, 0.047 mmol) treated with acetic acid, formaldehyde in 5 mL of EtOH was subjected to microwave heating at (90 °C, 200 watts) and gave the compound **7d** (14.0 mg, 80%) as a light yellow solid, R_f = 0.30 (25% ethyl acetate/hexane), **m.p**. 108–109 °C. **^{1}H-NMR (CDCl$_3$, 400 MHz):** δ 8.32 (2H, d, *J* = 8.6 Hz), 7. 69 (2H, d, *J* = 8.6 Hz), 7.28 (3H, overlap solvent residual peak), 7.19 (3H, q, *J* = 5.87 Hz), 7.09 (2H, t), 7.02 (2H, d, *J* = 7.4 Hz), 4.53 (1H, d, *J* = 13.5 Hz), 4.28 (2H, m), 4.10 (1H, d, *J* = 13.0 Hz), 3.82 (1H, d, *J* = 13.0 Hz), 3. 59 (1H, d, *J* = 13.4 Hz), 3.27 (2H, m), 2.94 (1H, d, *J* = 13.5 Hz) ppm. **^{13}C APT NMR (CDCl$_3$, 100 MHz):** δ 147.0, 136.6, 135.4, 129.0 (d), 128.7 (d), 128.6 (d), 128.5 (d), 128.4 (d), 127.8, 127.9, 124.8 (d), 66.0, 65.6, 53.6, 52.7, 51.1 ppm. **HRMS (ESI):** m/z calcd for $C_{23}H_{23}N_3O_4S$ $[M+H]^+$ 438.1488, found 438.1482.

2,4-bis(2-hydroxyethyl)-5-(4-nitrophenyl)-1,2,4-thiadiazinane 1,1-dioxide (7e)

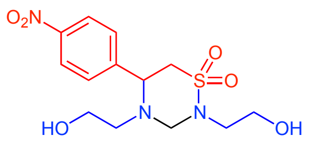

According to the general procedure (**1.3.3**), method III, *N*-(2hydroxyethyl)-2-(2-hydroxyethylamino)-2-(4-nitrophenyl)ethanesulfonamide (20.0 mg, 0.06 mmol) treated with acetic acid, formaldehyde in 5 mL of EtOH was subjected to microwave heating at (90 °C, 200 watts) and gave the compound **7e** (12.4 mg, 83%) as gold sticky oil, R_f = 0.56 (50% ethyl acetate/hexane). **^{1}H-NMR (CD_3OD, 400 MHz):** δ 8.16 (2H, d, *J* = 8.8 Hz), 7.61 (2H, d, *J* = 8.7 Hz), 4.36 (2H, s), 4.21 (1H, dd), 3.68 (2H, m), 3.42 (4H, m), 3.20 (4H, m), 2.46 (1H, m), 2.20 (1H, m) ppm. **^{13}C APT NMR (CD_3OD, 100 MHz):** δ 148.4, 130.3 (d), 125.1 (d), 70.9, 65.5, 62.2, 60.6, 53.0, 52.0, 51.5 ppm. **HRMS:** m/z calcd for $C_{13}H_{19}N_3O_6S$ $[M+H]^+$ 346.1053, found 346.1072.

2,4-dibutyl-5-phenyl-1,2,4-thiadiazinane 1,1-dioxide (7f)

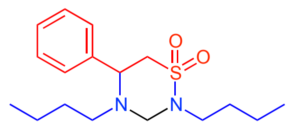

According to the general procedure (**1.3.1**), method I, *N*-butyl-2-(butylamino)-2-phenylethanesulfonamide (40.0 mg, 0.13 mmol) treated with DBU (50 mol%) in 3 mL DCM at 50 °C for 12 h gave the compound **7f** (16.6 mg, 40%) as a colorless oil, R_f= 0.54 (15% ethyl acetate/hexane), **^{1}H-NMR ($CDCl_3$, 400 MHz):** δ 7.33 (5H, m), 4.23 (1H, d, *J* = 12.9 Hz), 4.06 (1H, d, *J* = 12.9 Hz), 3.98 (1H, t, *J* = 7.1 Hz), 3.61 (1H, q, *J* = 7.1 Hz), 3.36 (2H, m), 3.10 (2H, m), 2.40 (1H, m), 2.03 (1H, m), 1.25 (8H, m), 0.96 (3H, t, *J* = 7.3 Hz), 0.75 (3H, t, *J* = 7.3 Hz) ppm. **^{13}C APT NMR ($CDCl_3$, 100 MHz):** δ 139.9, 128.4 (d), 127.4 (d), 92.1, 67.6, 64.9, 52.7, 48.4, 47.5, 30.8, 28.6, 20.1, 19.8, 13.7 (d) ppm. **HRMS (ESI):** m/z calcd for $C_{17}H_{28}N_3O_4S$ $[M+H]^+$ 325.1602, found 325.1705.

4-(2,4-dibenzyl-1,1-dioxido-1,2,4-thiadiazinan-5-yl)benzonitrile (7g)

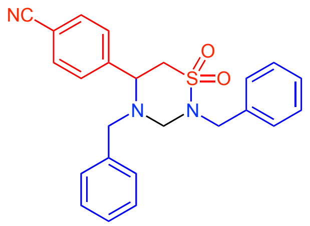

According to the general procedure (**1.3.3**), method III, *N*-(2hydroxyethyl)-2f-(2-hydroxyethylamino)-2-(4-nitrophenyl)ethanesulfonamide (20.0 mg, 0.06 mmol), which was synthesized following general procedure (**1.1.2**) followed by method III (**1.2.2**) (R_f= 0.5 in 50% ethyl acetate/hexane- isolated and confirmed by LC-MS only) was treated with acetic acid, formaldehyde in 5 mL of EtOH was subjected to microwave heating at (90 °C, 200 watts) and gave the compound **7g** (13 mg, 62%) as a white solid, R_f = 0.61 (25% ethyl acetate/hexane), **m.p.** 110–111 °C. **^{1}H-NMR ($CDCl_3$, 600 MHz):** δ 7.7 (2H, d, *J* = 7.8 Hz), 7.6 (2H, d, *J* = 8.7 Hz), 7.3 (3H, br s), 7.2 (3H, m), 7.1 (2H, br s), 7.0 (2H, d), 4.5 (2H, ddd), 4.1 (1H, d),3.8 (1H,d, *J* = 12.8 Hz), 3.6 (1H, d, *J* =13.5 Hz), 3.3 (2H, t), 2.9 (1H, d, *J* = 10.2 Hz) ppm. **^{13}C-NMR ($CDCl_3$, 100 MHz)** δ 144.9, 136.5, 135.3, 133.2, 129.0 (d), 128.7 (d), 128.6 (d), 128.5 (d), 128.3 (d), 127.9 (d), 127.8, 118.1, 112.9, 65.9,

65.1, 53.4, 52.6, 50.9 ppm. **HRMS (ESI):** m/z calcd for $C_{24}H_{23}N_3O_2S$ $[M+H]^+$ 418.1584, found 418.1583.

2,4-dibenzyl-5-(4-fluorophenyl)-1,2,4-thiadiazinane 1,1-dioxide (7h)

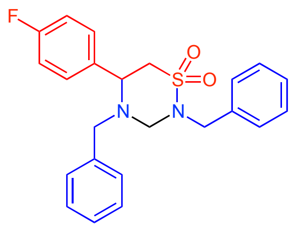

According to the general procedure (**1.3.3**), method III, *N*-(2hydroxyethyl)-2-(2-hydroxyethylamino)-2-(4-nitrophenyl)ethanesulfonamide (20.0 mg, 0.06 mmol) which was synthesized following general procedure (**1.1.2**) followed by method III (**1.2.2**) (R_f = 0.35 in 25% ethyl acetate/hexane- isolated and confirmed by LC-MS only) was treated with acetic acid, formaldehyde in 5 mL of EtOH was subjected to microwave heating at (90 °C, 200 watts) and gave the compound **7h** (16 mg, 80%) as white powder, R_f = 0.45 (10% ethyl acetate/hexane), **^{1}H-NMR ($CDCl_3$, 600 MHz):** δ 7.53-7.46 (2H, m), 7. 31-7.28 (3H, m), 7.25-5.16 (5H, m), 7.15-5.11 (2H, m), 7.02 (2H, d, J = 7.8 Hz), 4.55 (1H, d, *J* = 13.2 Hz), 4.27 (1H, d, J = 13.2 Hz), 4.17 (1H, t, *J* = 7.2 Hz), 4.08 (1H, d, *J* = 12.6 Hz), 3.80 (1H, d, *J* = 13.8 Hz), 3.68 (1H, d, J = 13.2 Hz), 3.30 (2H, d, *J* = 7.2 Hz), 2.90 (1H, d, J = 13.8 Hz) pm. **^{13}C NMR ($CDCl_3$ 100 MHz** δ 163.6, 162.7, 137.1, 135.6, 129.2 (d), 129.1 (d), 129.0 (d), 128.7 (d), 128.4 (d), 127.7, 127.5, 116.4, 66.0, 64.8, 53.2, 53.1, 50.1 (d) ppm. **HRMS (ESI):** m/z calcd for $C_{23}H_{23}N_2O_2SF$ $[M+H]^+$ 411.1537, found 411.1534.

2,4-dibenzyl-5-(4a,8a-dihydronaphthalen-2-yl)-1,2,4-thiadiazinane 1,1-dioxide (7i)

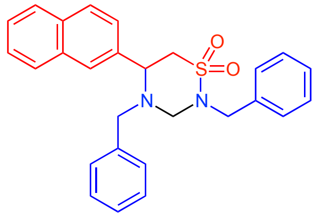

According to the general procedure (**1.3.3**), method III, *N*-(2hydroxyethyl)-2-(2-hydroxyethylamino)-2-(4-nitrophenyl)ethanesulfonamide (20.0 mg, 0.06 mmol) which was synthesized following general procedure (**1.1.2**) followed by method III (**1.2.2**) (R_f = 0.4 in 25% ethyl acetate/hexane- isolated and confirmed by LC-MS only) was treated with acetic acid, formaldehyde in 5 mL of EtOH was subjected to microwave heating at (90 °C, 200 watts) and gave the compound **7i** (16 mg, 75%) as white viscous oil, R_f = 0.5 (10% ethyl acetate/hexane). **^{1}H-NMR ($CDCl_3$, 600 MHz):** δ 8.56 (1H, s), 7. 91 (1H, d, *J* = 7.8 Hz), 7.71 (1H, d, J = 7.2Hz), 7.67 (1H, t, *J* = 7.8 Hz), 7.60 (1H, t, *J* = 7.2 Hz), 7.56 (1H, t, *J*= 7.8Hz) 7.29-7.17 (6H, m), 7.13 (2H, d, J = 7.2 Hz), 6.96 (2H, d, J = 6 Hz), 5.07-4.96 (1H, m), 4.52 -4.42 (2H, m), 4.25 (1H, d, *J* = 12.6 Hz), 3.90 (1H, d, *J* = 13.2 Hz), 3.78-3.67 (2H, m), 3.45-3.38 (1H, m), 3.01 (1H, d, J = 13.8 Hz), ppm. **^{13}C NMR ($CDCl_3$ 100 MHz):** δ 137.2, 135.7, 134.7, 130.9, 129.4 (d), 129.2 (d), 129.1 (d), 129.0 (d), 128.5 (d), 128.4 (d), 127.8 (d), 127.4 (d), 126.7 (d), 126.3, 66.3 (d), 52.1 (d) ppm. **HRMS:** m/z calcd for $C_{27}H_{26}N_2O_2S$ $[M+H]^+$ 443.1788, found 443.1785.

1.5 References

1. G.-F. Zha, G. A. L. Bare, J. Leng, Z.-P. Shang, Z. Luo and H.-L. Qin, *Adv. Synth. Catal.*, **2017**, *359*, 3237-3242.
2. P. K. Chinthakindi, K. B. Govender, A. S. Kumar, H. G. Kruger, T. Govender, T. Naicker and P. I. Arvidsson, *Org. Lett.*, **2017**, *19*, 480-483.

Spectral data

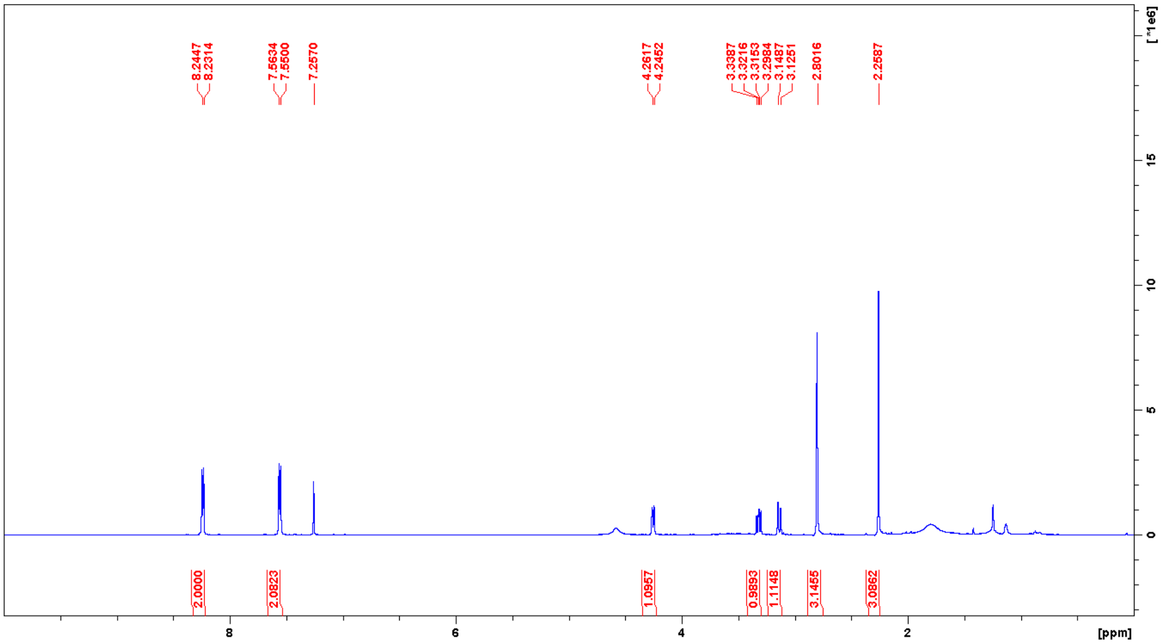

Figure S1. ^{1}H NMR (400 MHz, $CDCl_3$) of ***N*-methyl-2-(methylamino)-2-(4-nitrophenyl)ethanesulfonamide (6a)**.

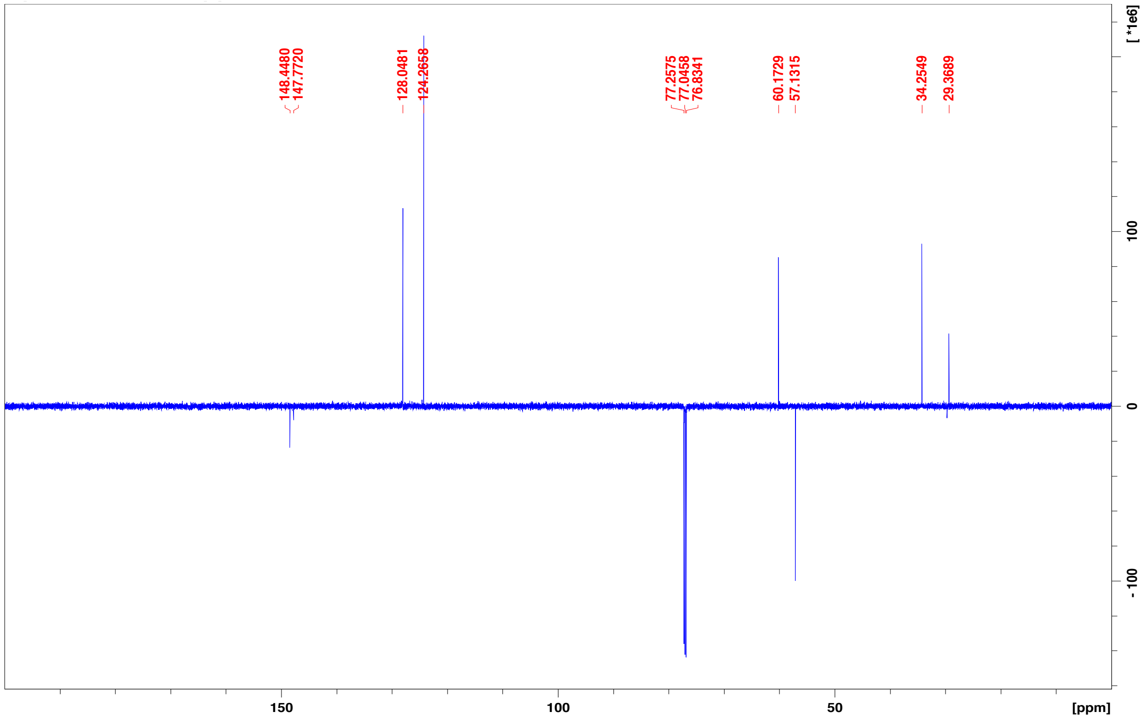

Figure S2. ^{13}C NMR (100 MHz, $CDCl_3$) of ***N*-methyl-2-(methylamino)-2-(4-nitrophenyl)ethanesulfonamide (6a)**.

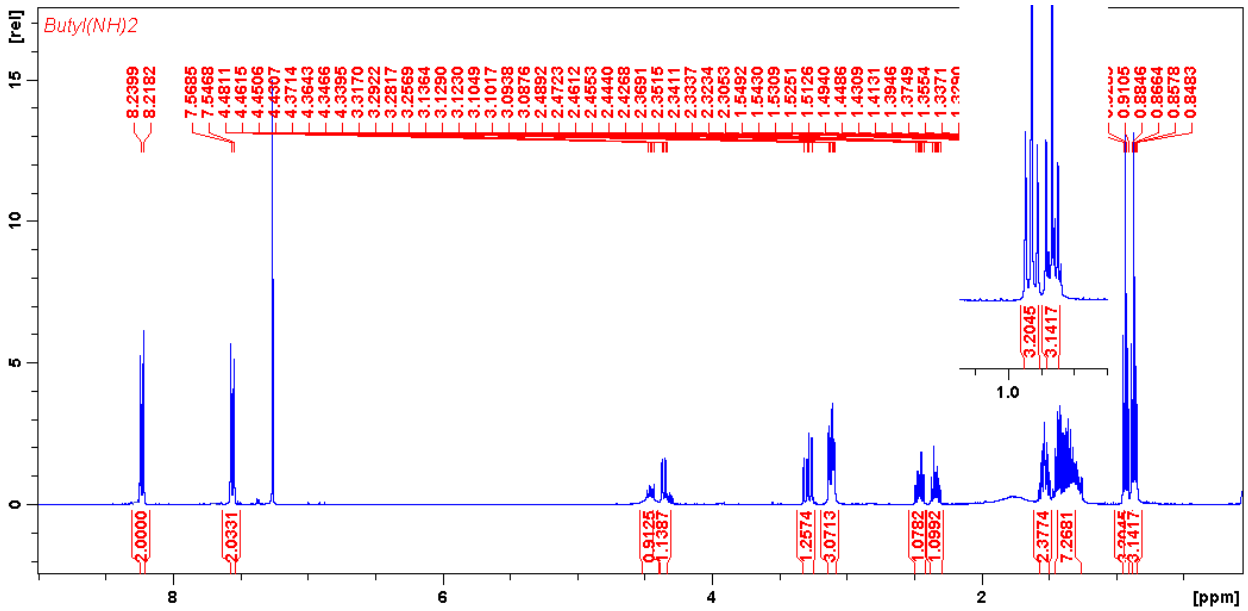

Figure S3. ^{1}H NMR (400 MHz, $CDCl_3$) of ***N*-butyl-2-(butylamino)-2-(4-nitrophenyl)ethanesulfonamide (6b)**.

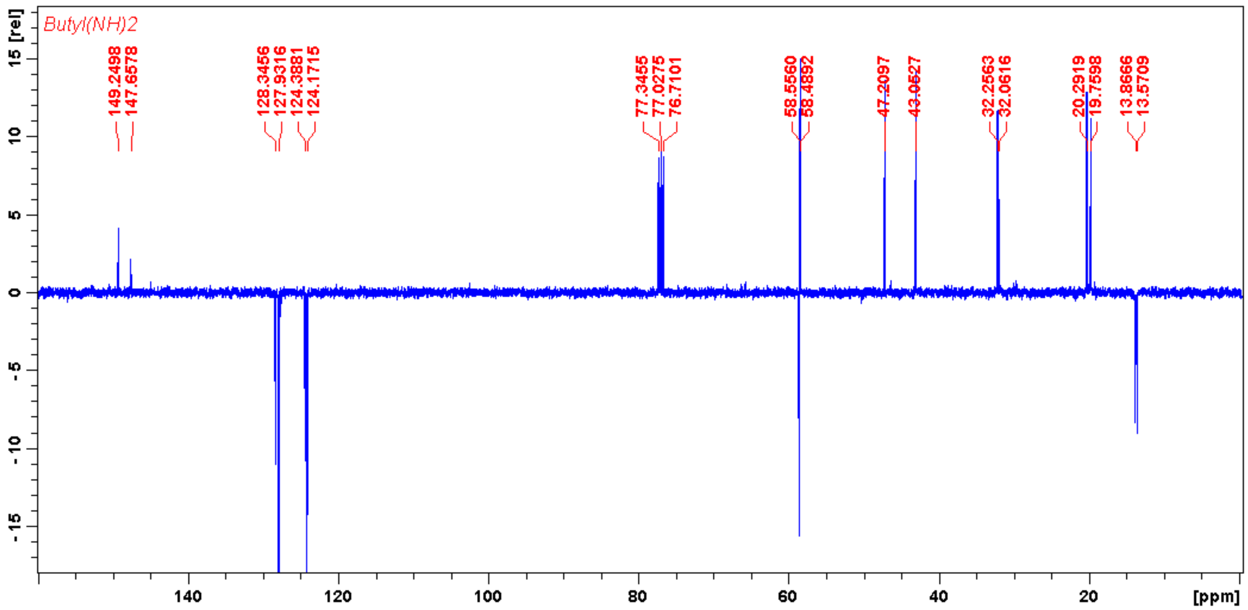

Figure S4. ^{13}C NMR (100 MHz, $CDCl_3$) of ***N*-butyl-2-(butylamino)-2-(4-nitrophenyl)ethanesulfonamide (6b)**.

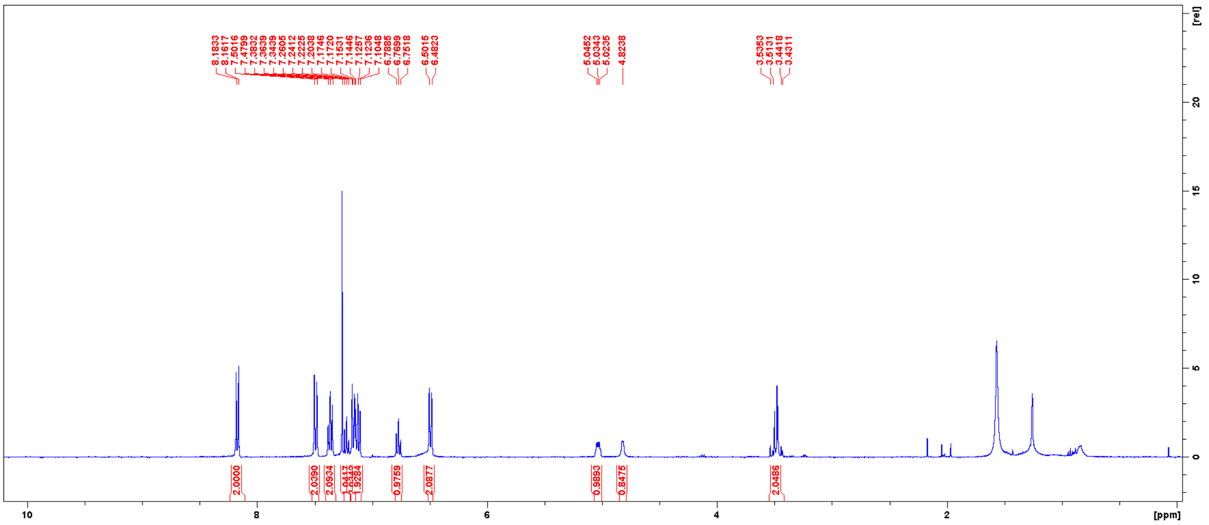

Figure S5. ^{1}H NMR (400 MHz, $CDCl_3$) of **2-(4-nitrophenyl)-*N*-phenyl-2-(phenylamino)ethanesulfonamide** (**6c**).

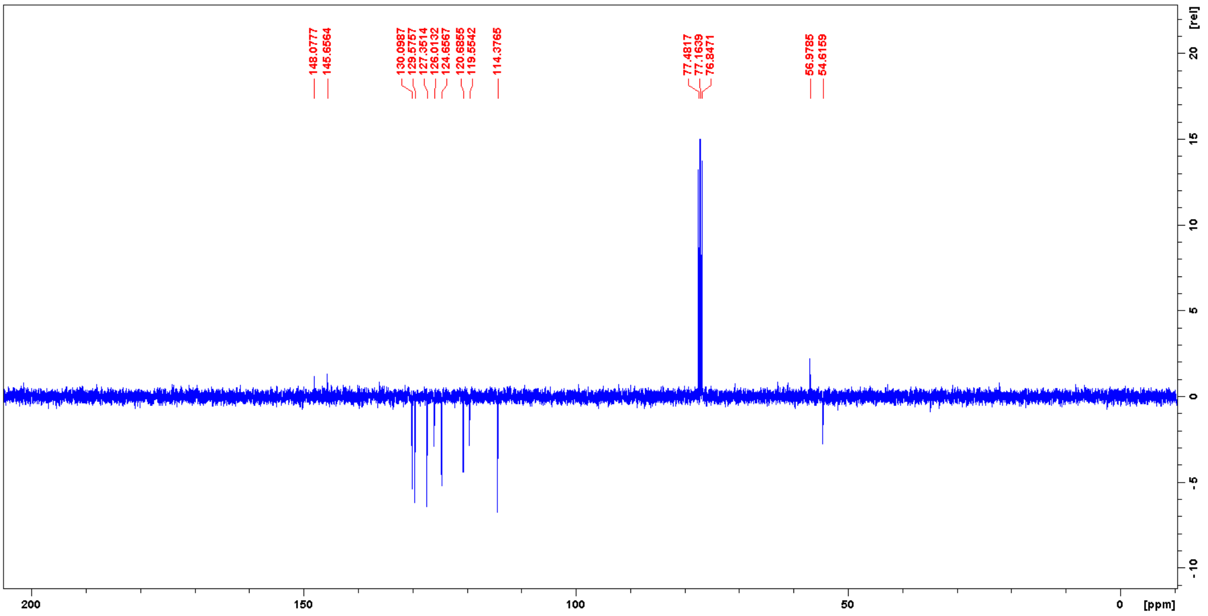

Figure S6. ^{13}C NMR (100 MHz, $CDCl_3$) of **2-(4-nitrophenyl)-*N*-phenyl-2-(phenylamino)ethanesulfonamide** (**6c**).

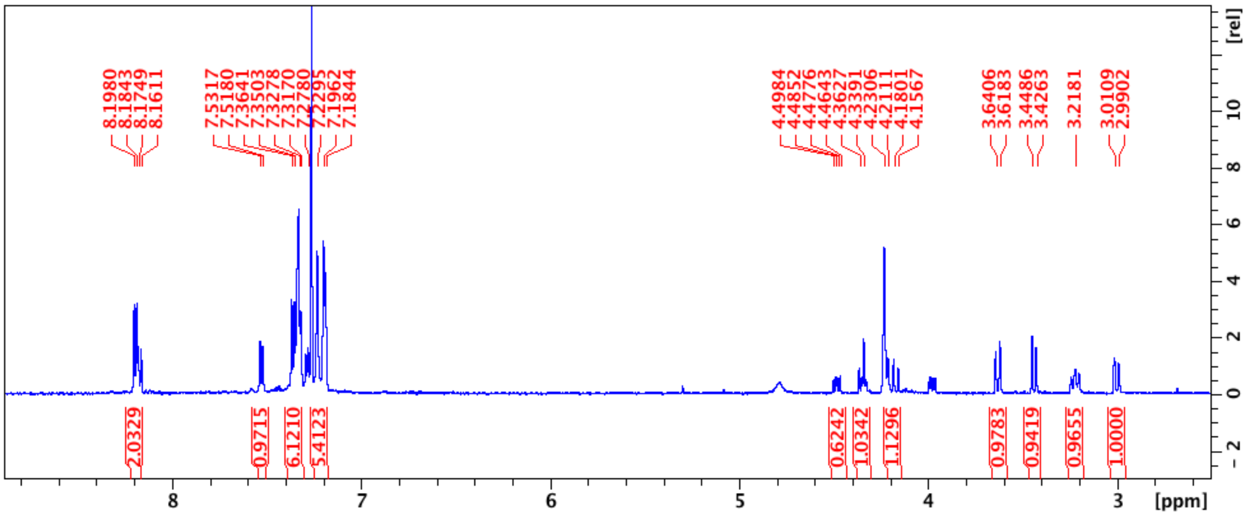

Figure S7. ^{1}H NMR (400 MHz, $CDCl_3$) of ***N*-benzyl-2-(benzylamino)-2-(4-nitrophenyl)ethanesulfonamide (6d)**.

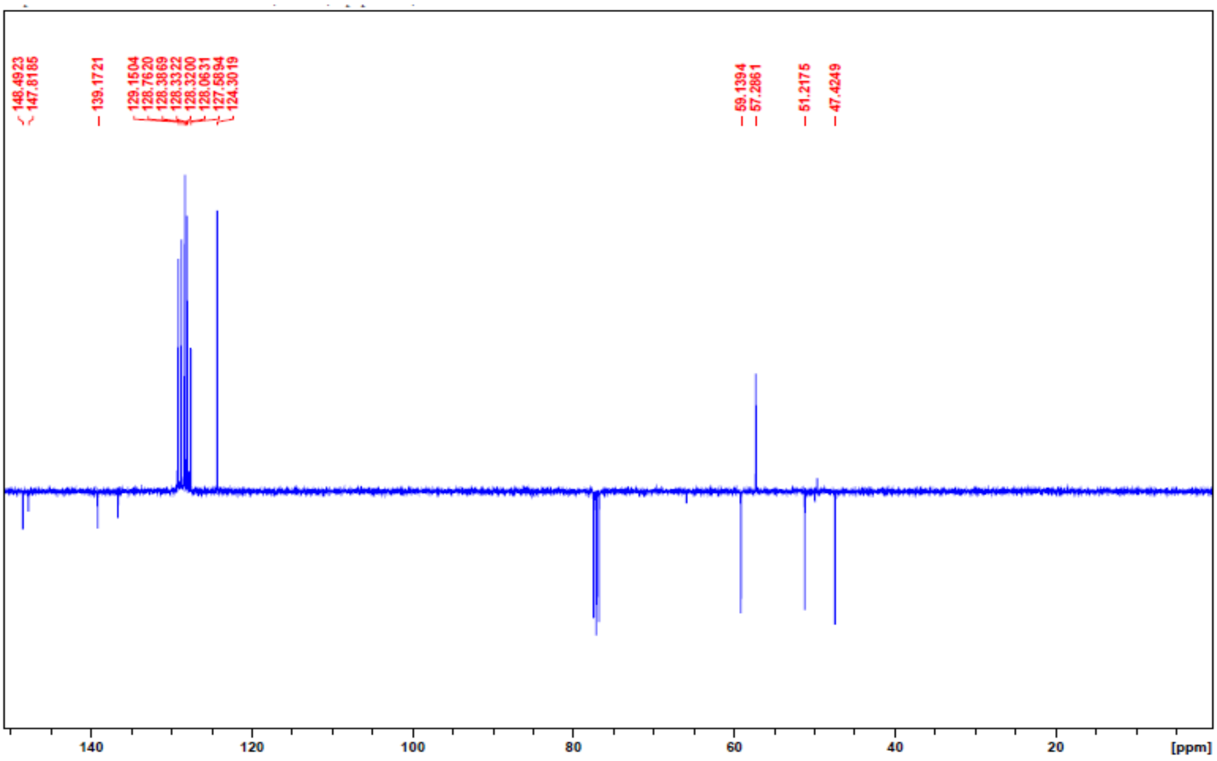

Figure S8. ^{13}C NMR (100 MHz, $CDCl_3$) of ***N*-benzyl-2-(benzylamino)-2-(4-nitrophenyl)ethanesulfonamide (6d)**.

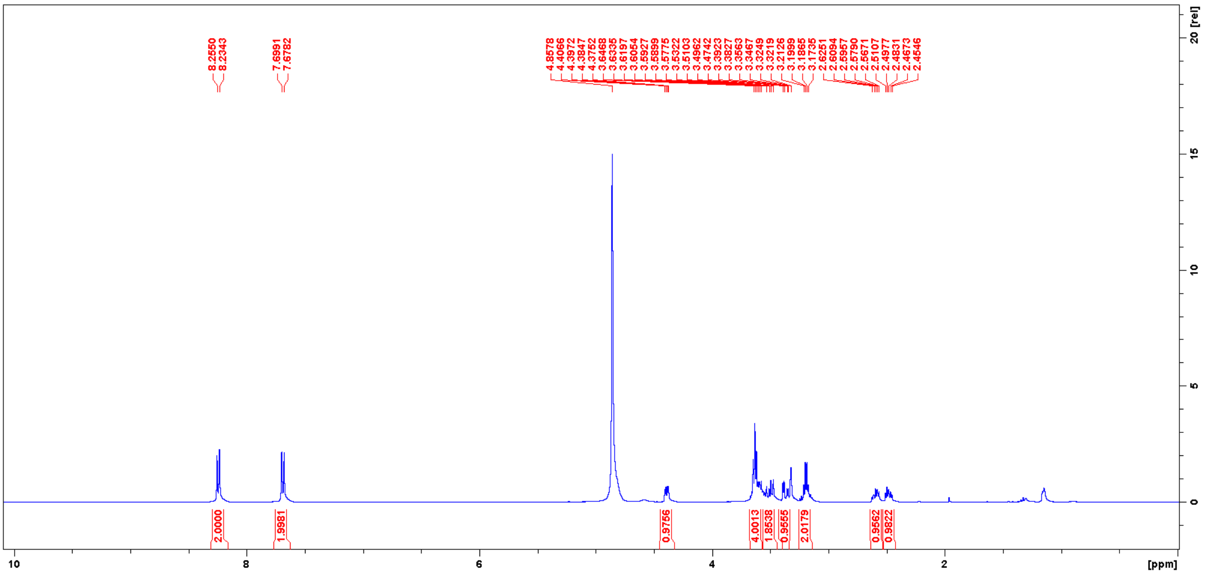

Figure S9. ^{1}H NMR (600 MHz, MeOD) of ***N*-(2-hydroxyethyl)-2-(2-hydroxyethylamino)-2-(4-nitrophenyl)ethanesulfonamide** (**6e**).

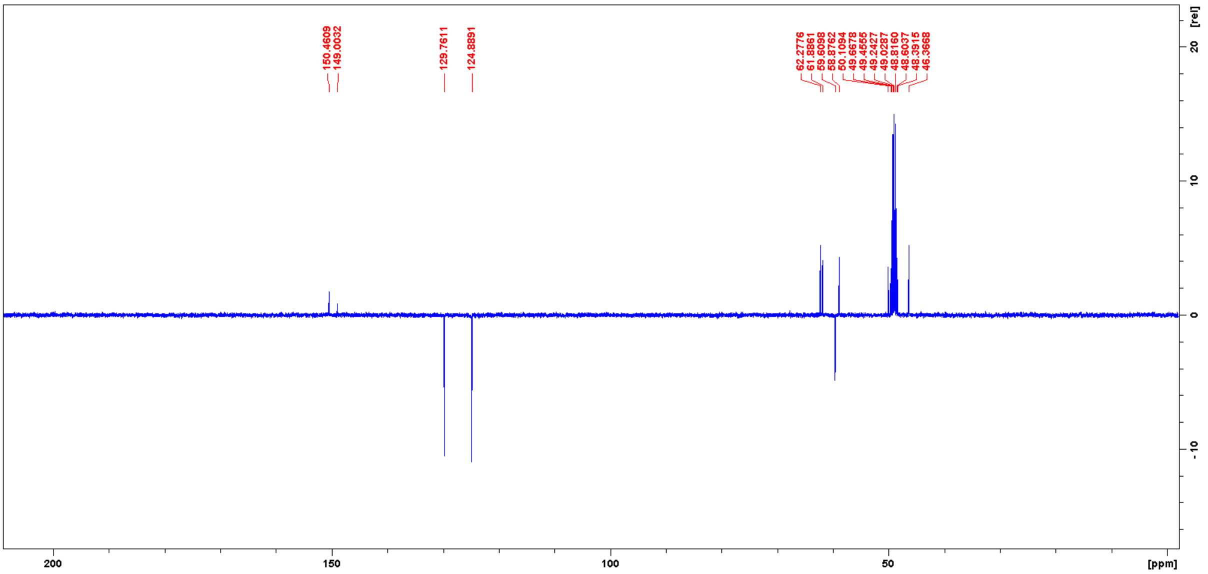

Figure S10. ^{13}C NMR (100 MHz, MeOD) of ***N*-(2-hydroxyethyl)-2-(2-hydroxyethylamino)-2-(4-nitrophenyl)ethanesulfonamide** (**6e**).

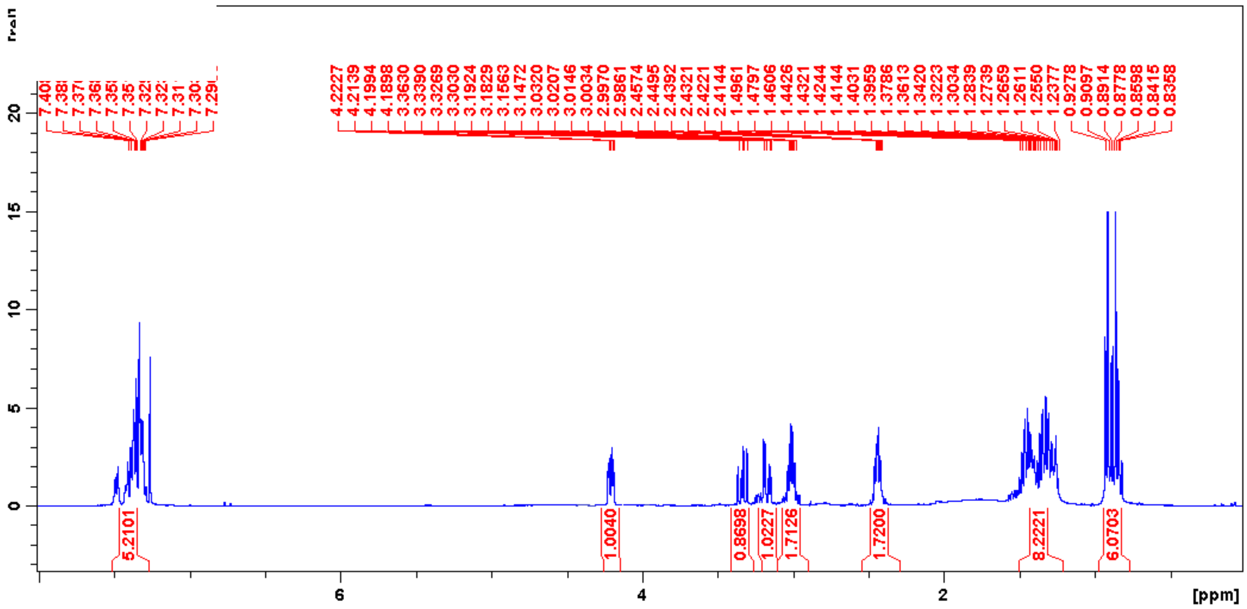

Figure S11. ^{1}H NMR (400 MHz, $CDCl_3$) of ***N*-butyl-2-(butylamino)-2-phenylethanesulfonamide** (**6f**).

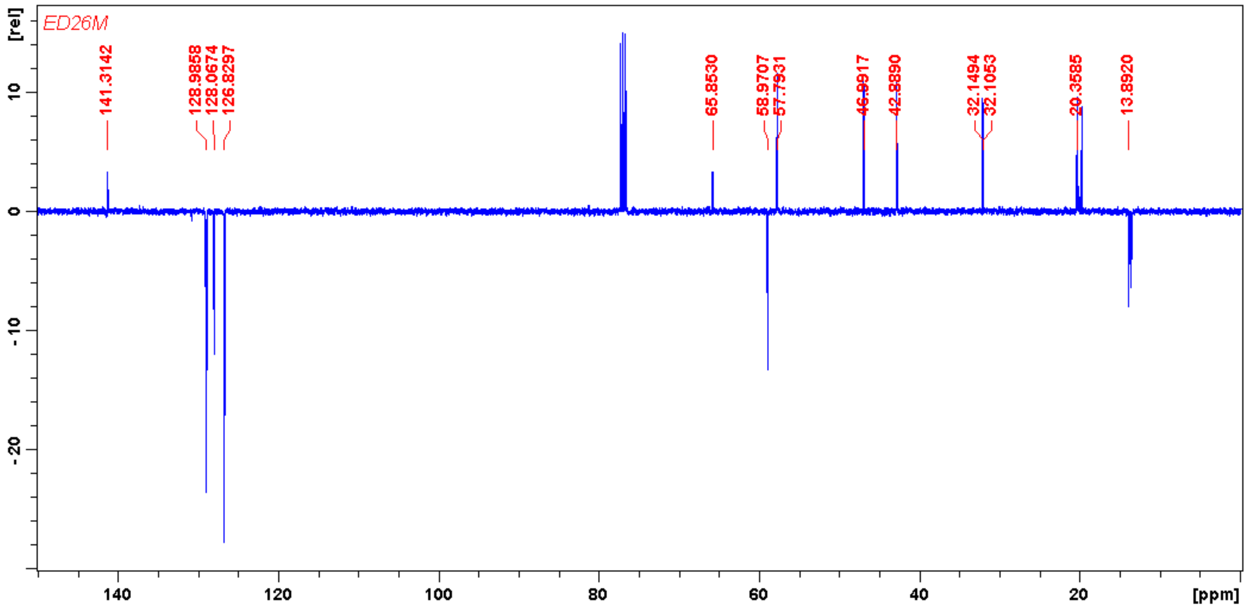

Figure S12. ^{13}C NMR (100 MHz, $CDCl_3$) of ***N*-butyl-2-(butylamino)-2-phenylethanesulfonamide** (**6f**).

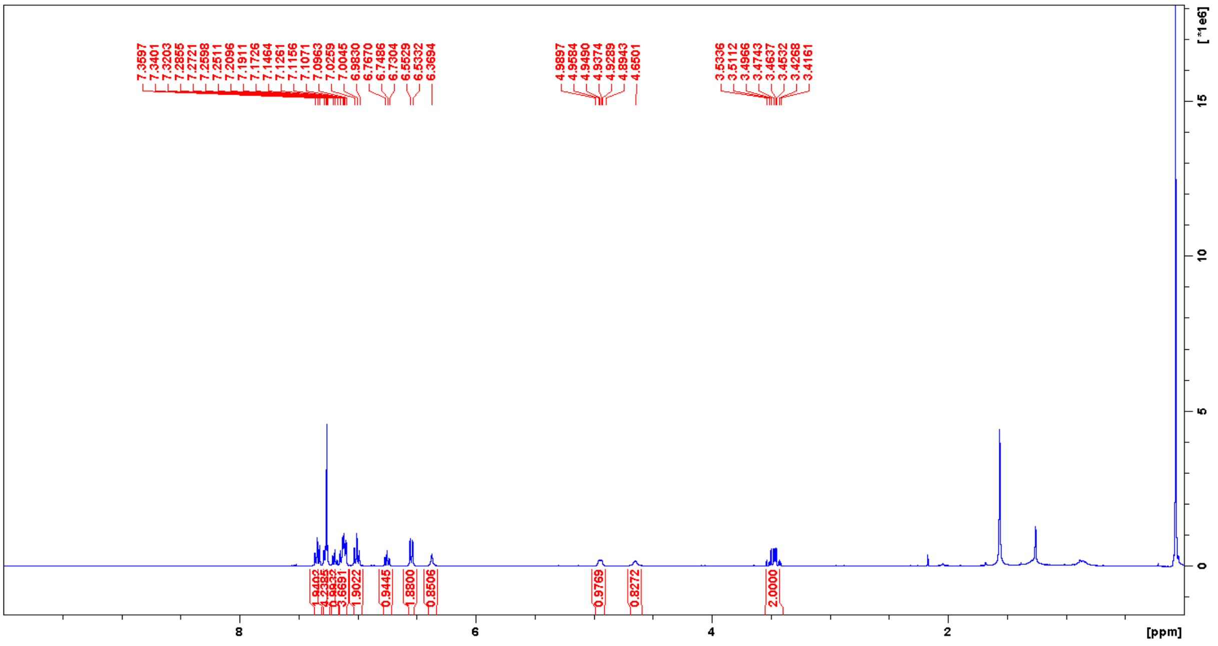

Figure S13. ^{1}H NMR (600 MHz, $CDCl_3$) of **2-(4-fluorophenyl)-*N*-phenyl-2-(phenylamino)ethanesulfonamide (6g)**

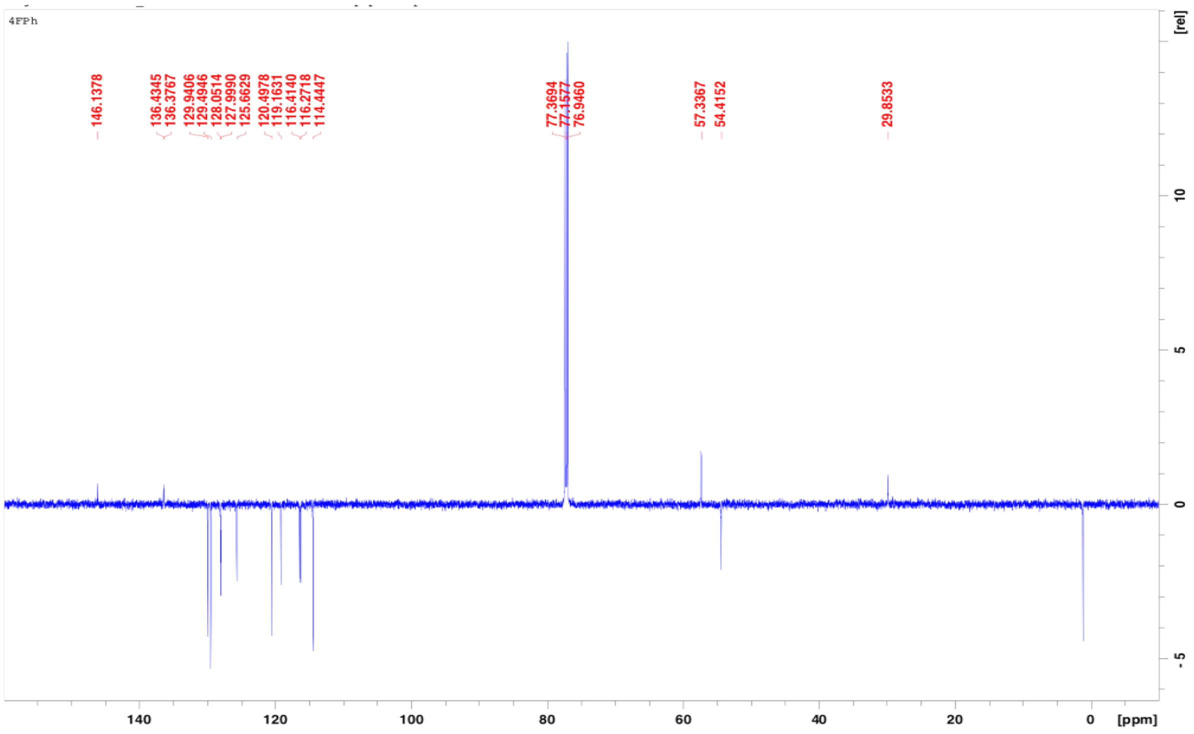

Figure S14. ^{13}C NMR (100 MHz, $CDCl_3$) of **2-(4-fluorophenyl)-*N*-phenyl-2-(phenylamino)ethanesulfonamide (6g)**

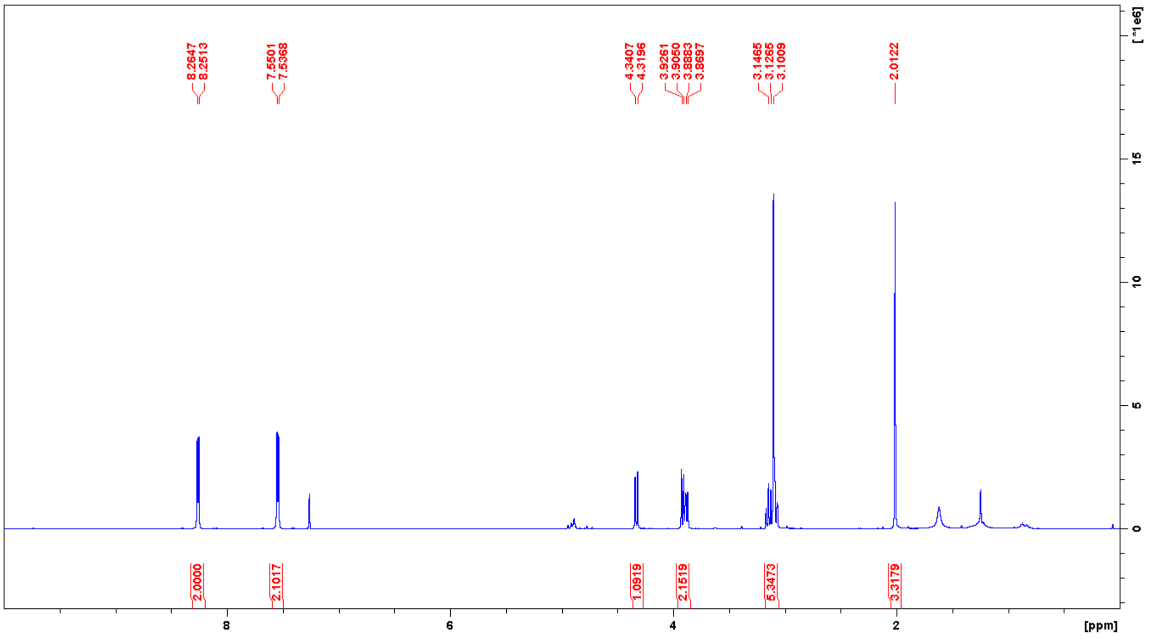

Figure S15. ^{1}H NMR (400 MHz, $CDCl_3$) of **2,4-dimethyl-5-(4-nitrophenyl)-1,2,4-thiadiazinane 1,1-dioxide (7a)**

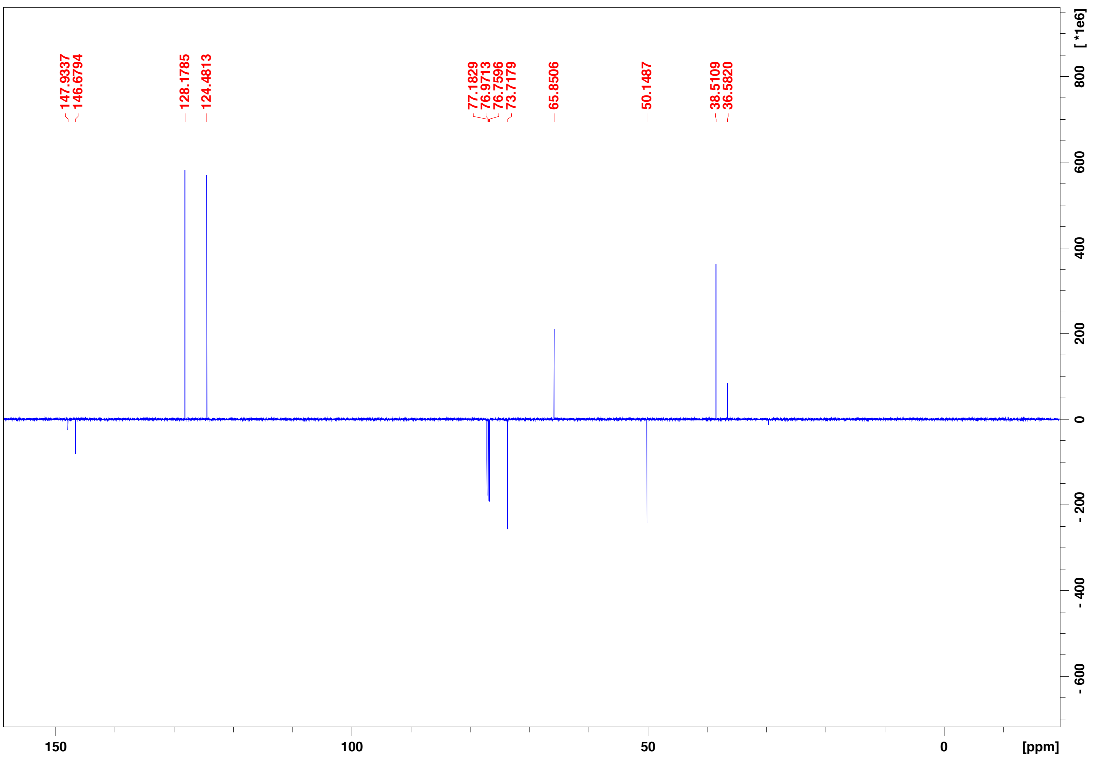

Figure S16. ^{13}C NMR (100 MHz, $CDCl_3$) of **2,4-dimethyl-5-(4-nitrophenyl)-1,2,4-thiadiazinane 1,1-dioxide (7a)**

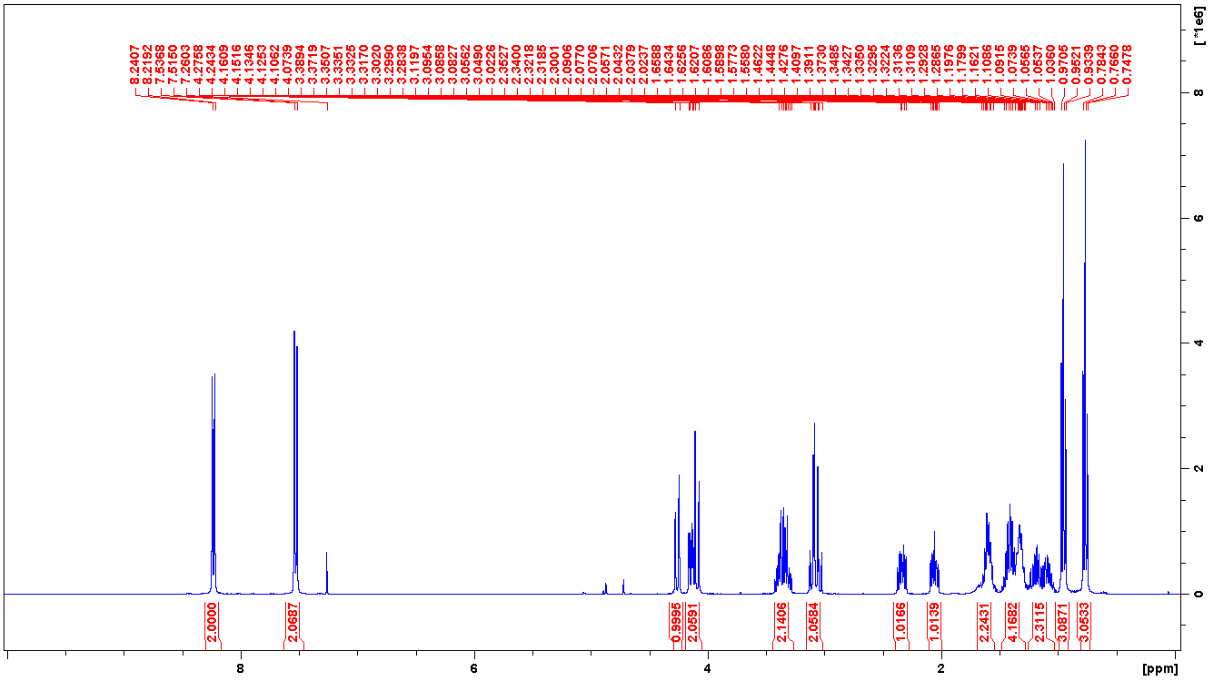

Figure S17. ^{1}H NMR (400 MHz, $CDCl_3$) of **2,4-dibutyl-5-(4-nitrophenyl)-1,2,4-thiadiazinane 1,1-dioxide (7b)**

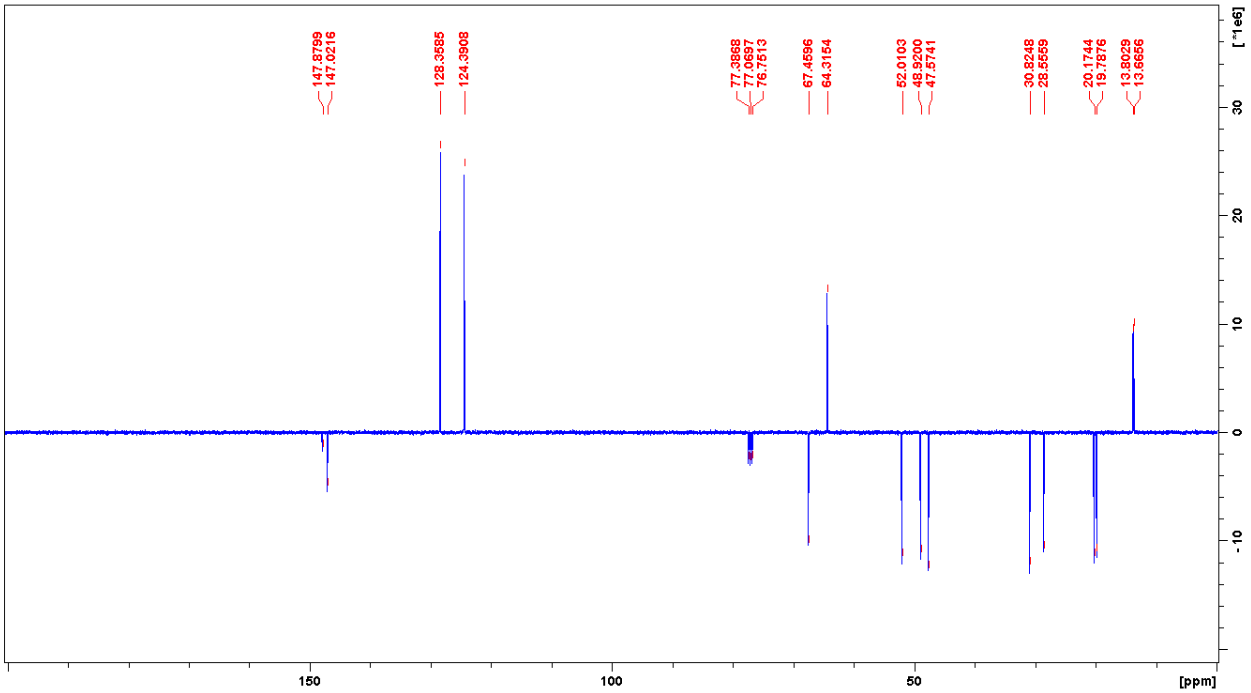

Figure S18. ^{13}C NMR (100 MHz, $CDCl_3$) of **2,4-dibutyl-5-(4-nitrophenyl)-1,2,4-thiadiazinane 1,1-dioxide (7b)**

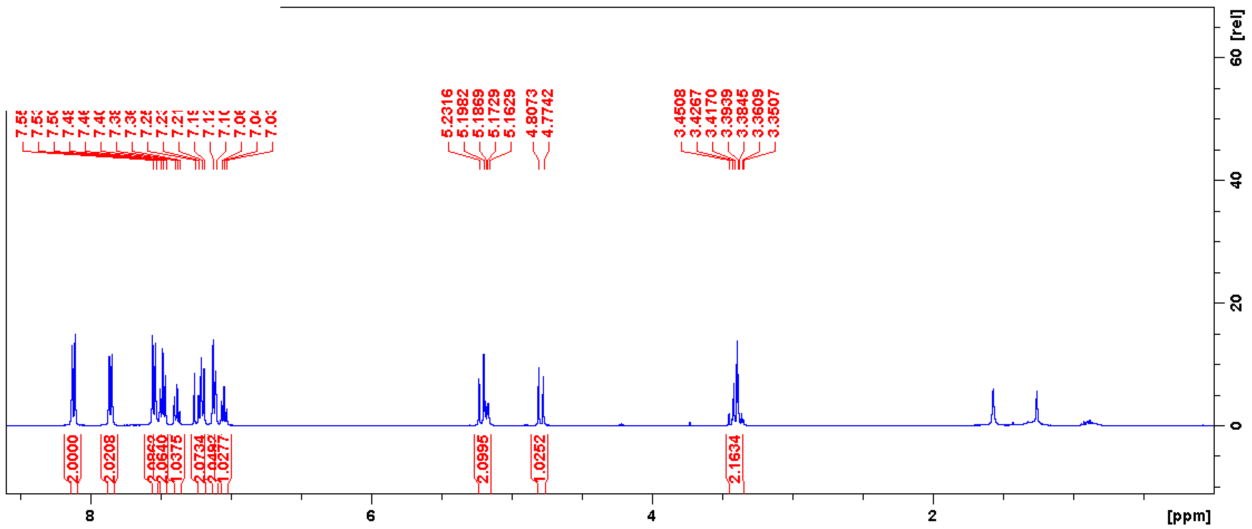

Figure S19. ^{1}H NMR (400 MHz, $CDCl_3$) of **5-(4-nitrophenyl)-2,4-diphenyl-1,2,4-thiadiazinane 1,1-dioxide (7c)**

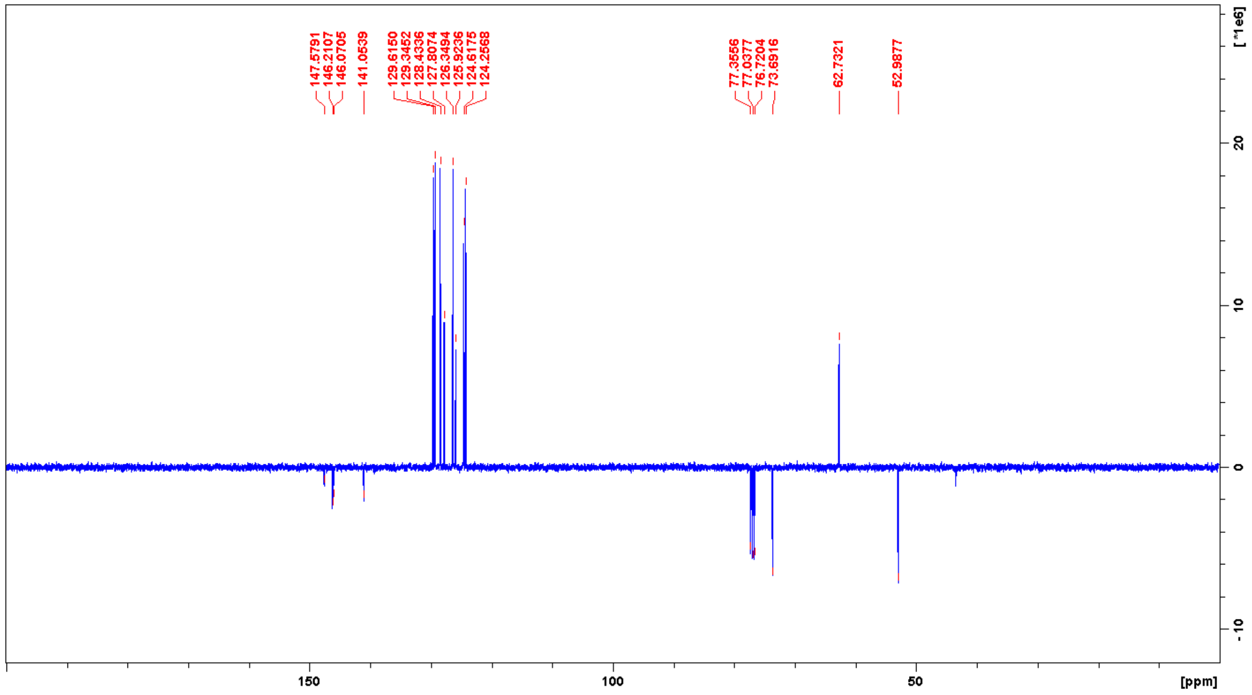

Figure S20. ^{13}C NMR (100 MHz, $CDCl_3$) of **5-(4-nitrophenyl)-2,4-diphenyl-1,2,4-thiadiazinane 1,1-dioxide (7c)**

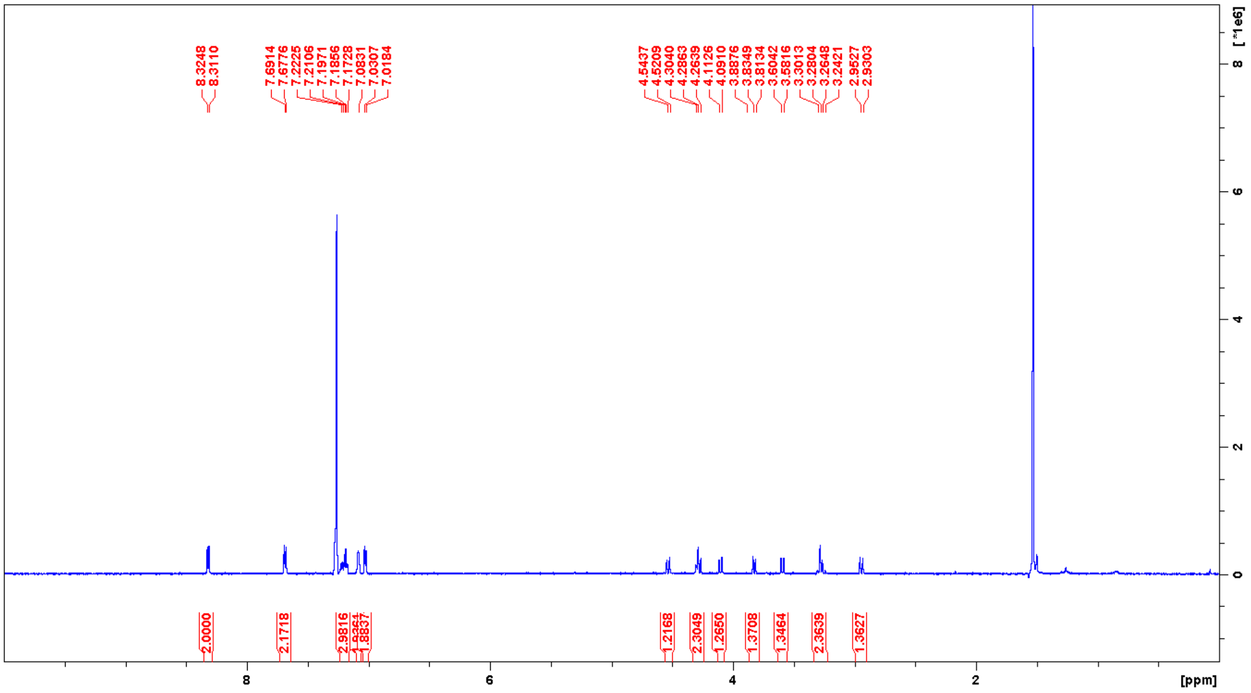

Figure S21. ^{1}H NMR (400 MHz, $CDCl_3$) of **2,4-dibenzyl-5-(4-nitrophenyl)-1,2,4-thiadiazinane 1,1-dioxide (7d)**

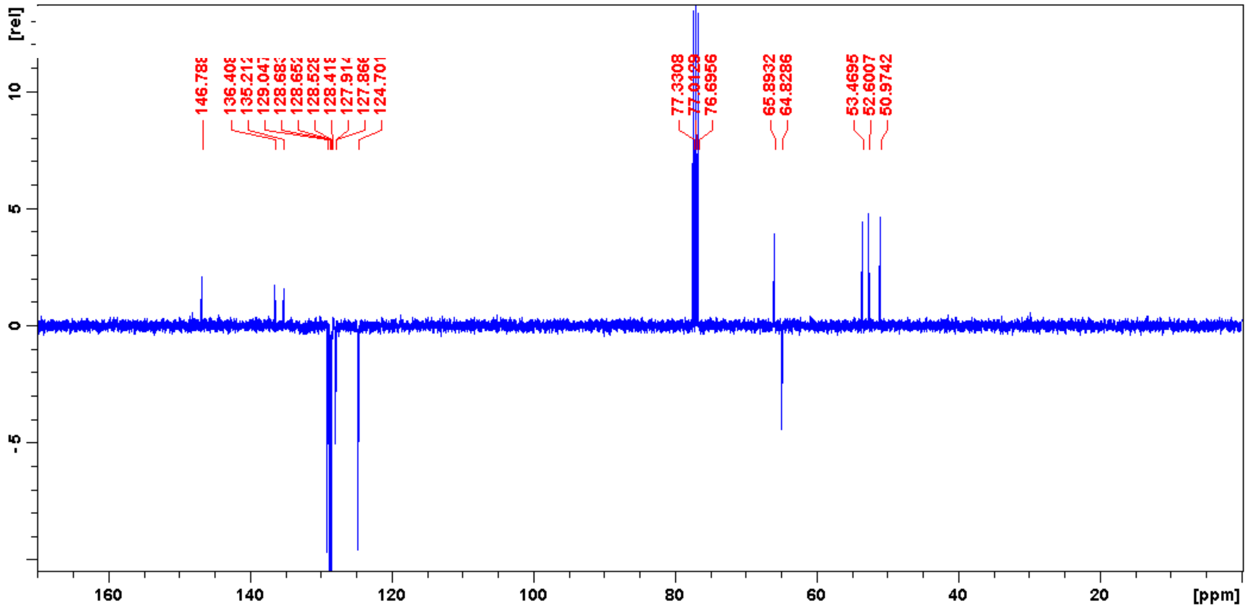

Figure S22. ^{13}C NMR (400 MHz, $CDCl_3$) of **2,4-dibenzyl-5-(4-nitrophenyl)-1,2,4-thiadiazinane 1,1-dioxide (7d)**

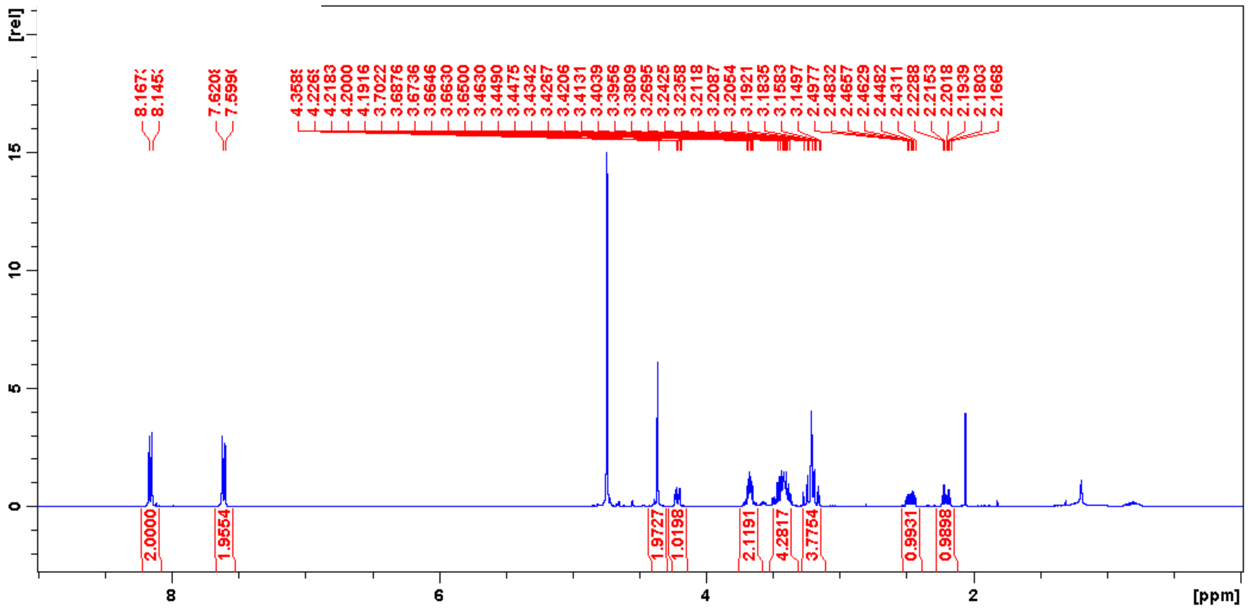

Figure S23. ^{1}H NMR (400 MHz, MeOD) of **2,4-bis(2-hydroxyethyl)-5-(4-nitrophenyl)-1,2,4-thiadiazinane 1,1-dioxide (7e)**

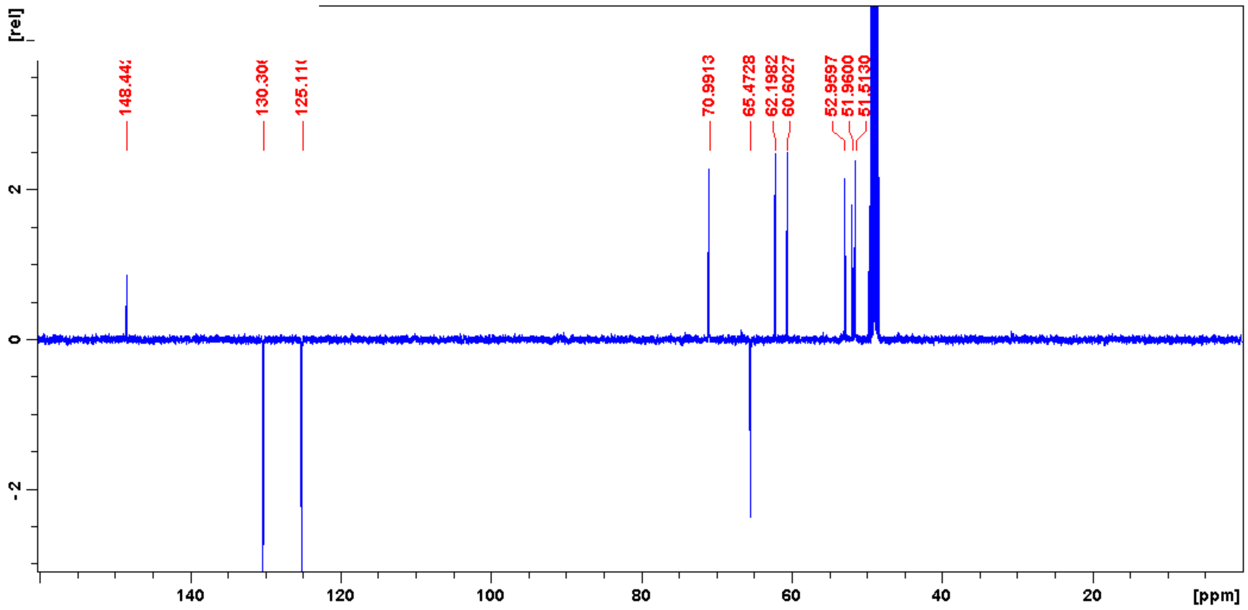

Figure S24. ^{13}C NMR (100 MHz, MeOD) of **2,4-bis(2-hydroxyethyl)-5-(4-nitrophenyl)-1,2,4-thiadiazinane 1,1-dioxide (7e)**

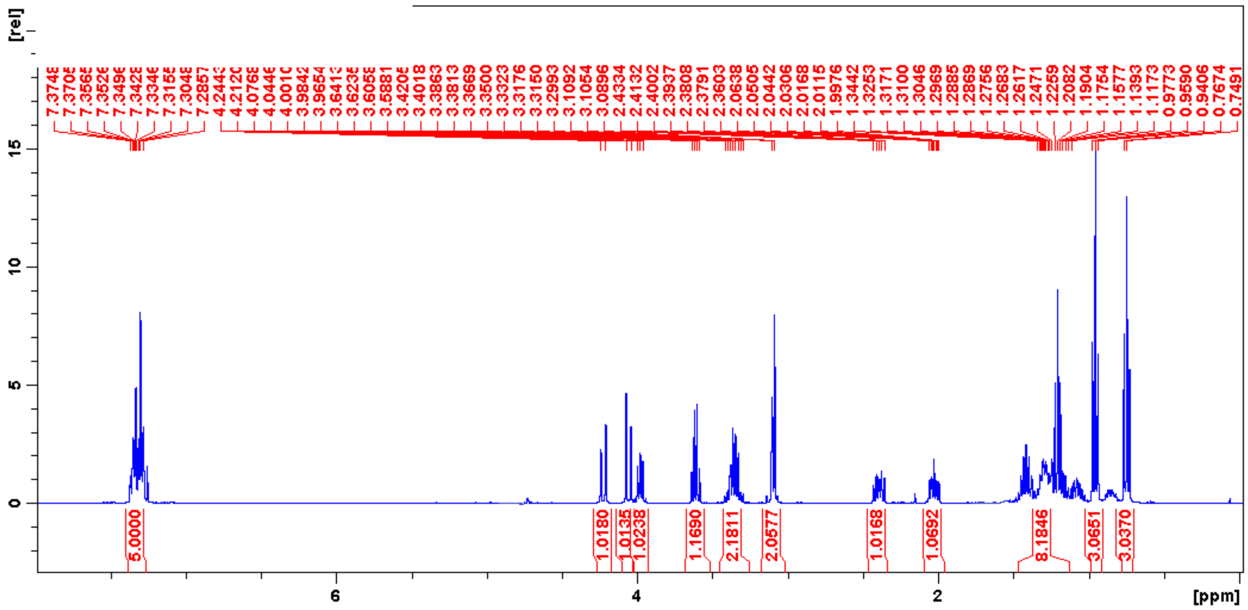

Figure S25. [1] H NMR (400 MHz, $CDCl_3$) of **2,4-dibutyl-5-phenyl-1,2,4-thiadiazinane 1,1-dioxide (7f)**

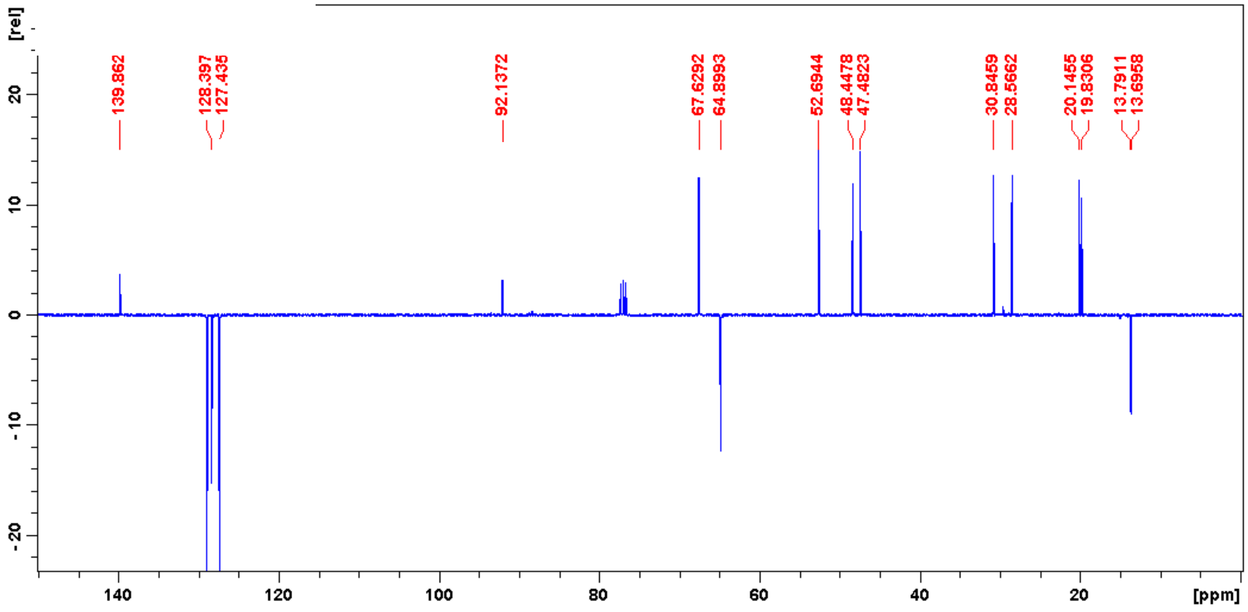

Figure S26. ^{13}C NMR (100 MHz, $CDCl_3$) of **2,4-dibutyl-5-phenyl-1,2,4-thiadiazinane 1,1-dioxide (7f)**

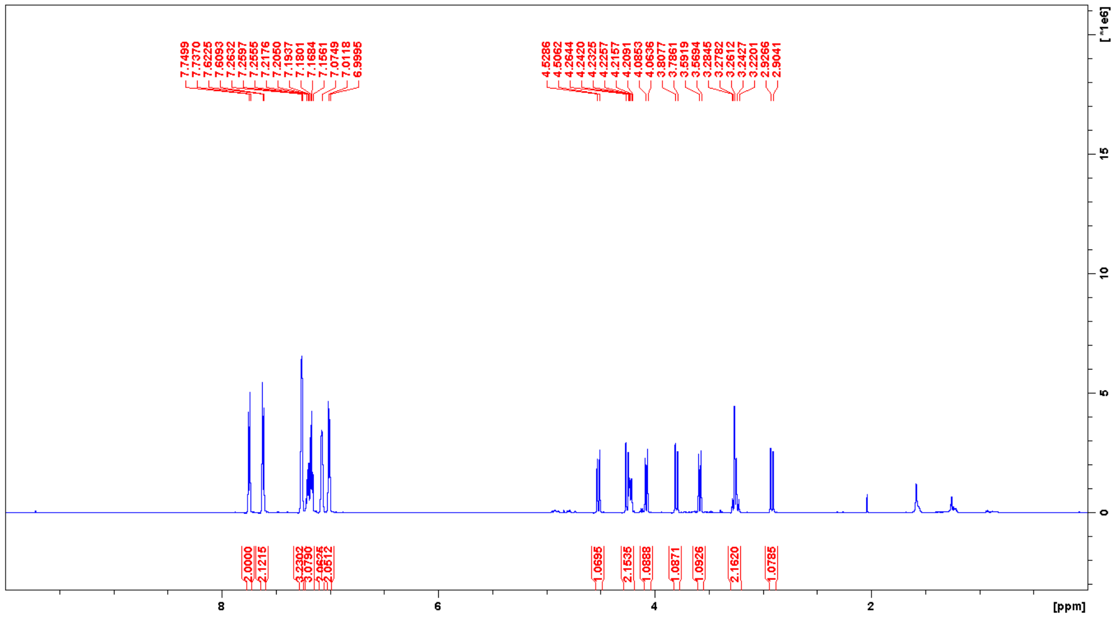

Figure S27. ^{1}H NMR (400 MHz, $CDCl_3$) of **4-(2,4-dibenzyl-1,1-dioxido-1,2,4-thiadiazinan-5-yl)benzonitrile (7g)**

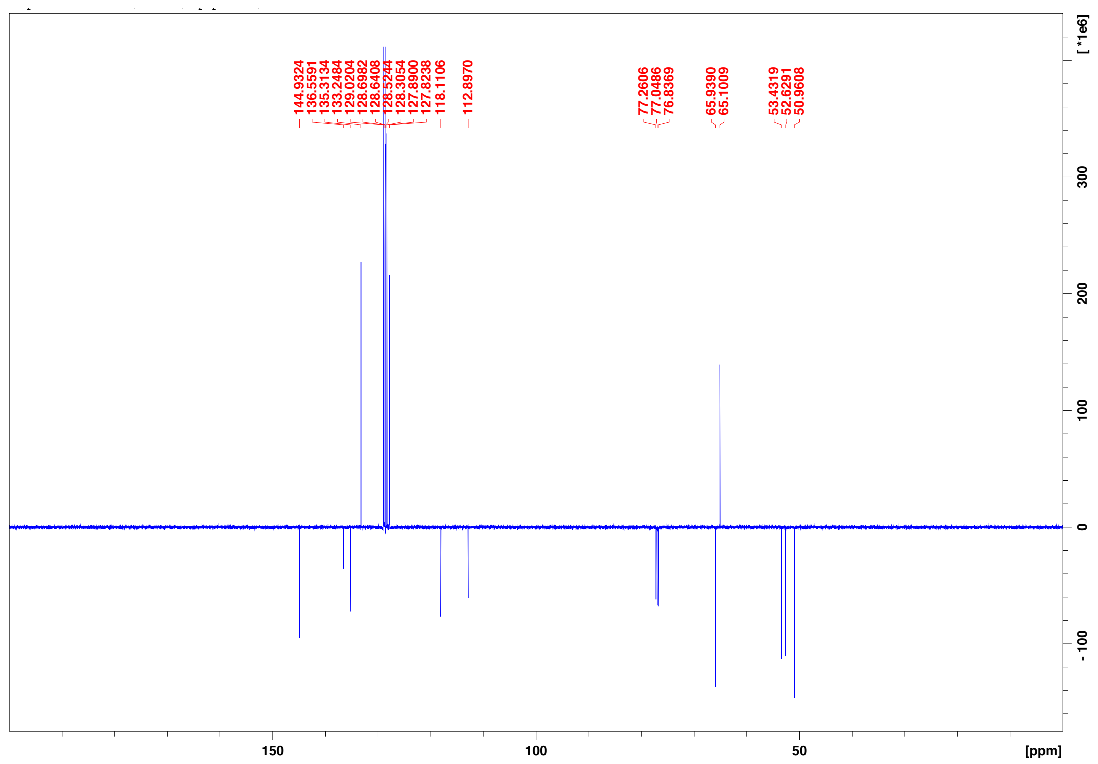

Figure S28. ^{13}C NMR (100 MHz, $CDCl_3$) of **4-(2,4-dibenzyl-1,1-dioxido-1,2,4-thiadiazinan-5-yl)benzonitrile (7g)**

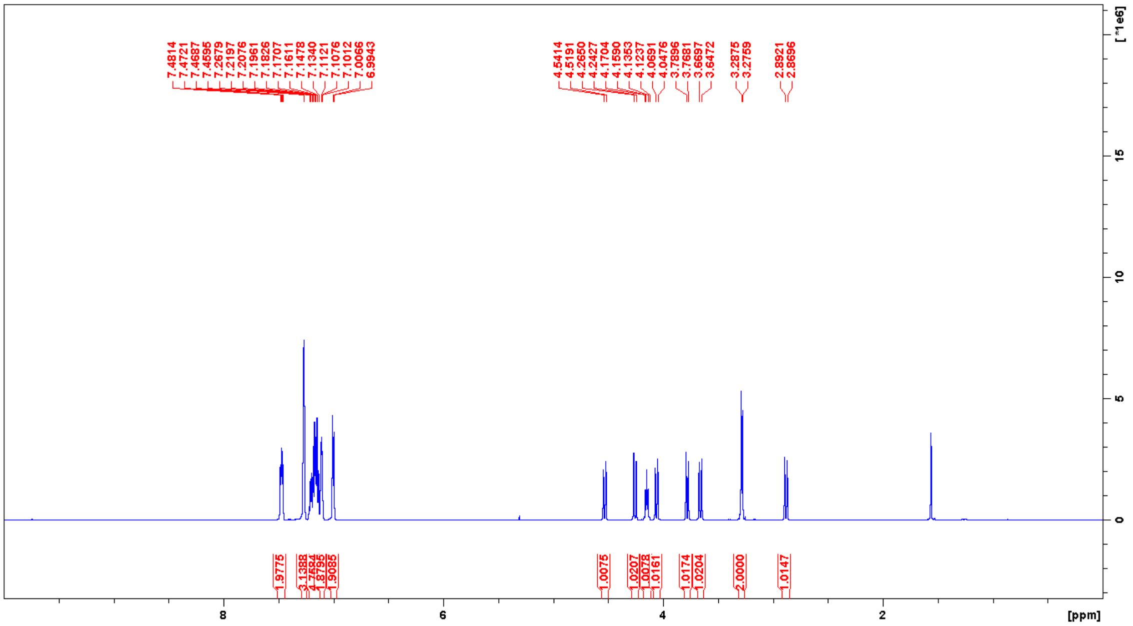

Figure S29. [1] H NMR (400 MHz, $CDCl_3$) of **2,4-dibenzyl-5-(4-fluorophenyl)-1,2,4-thiadiazinane 1,1-dioxide (7h)**

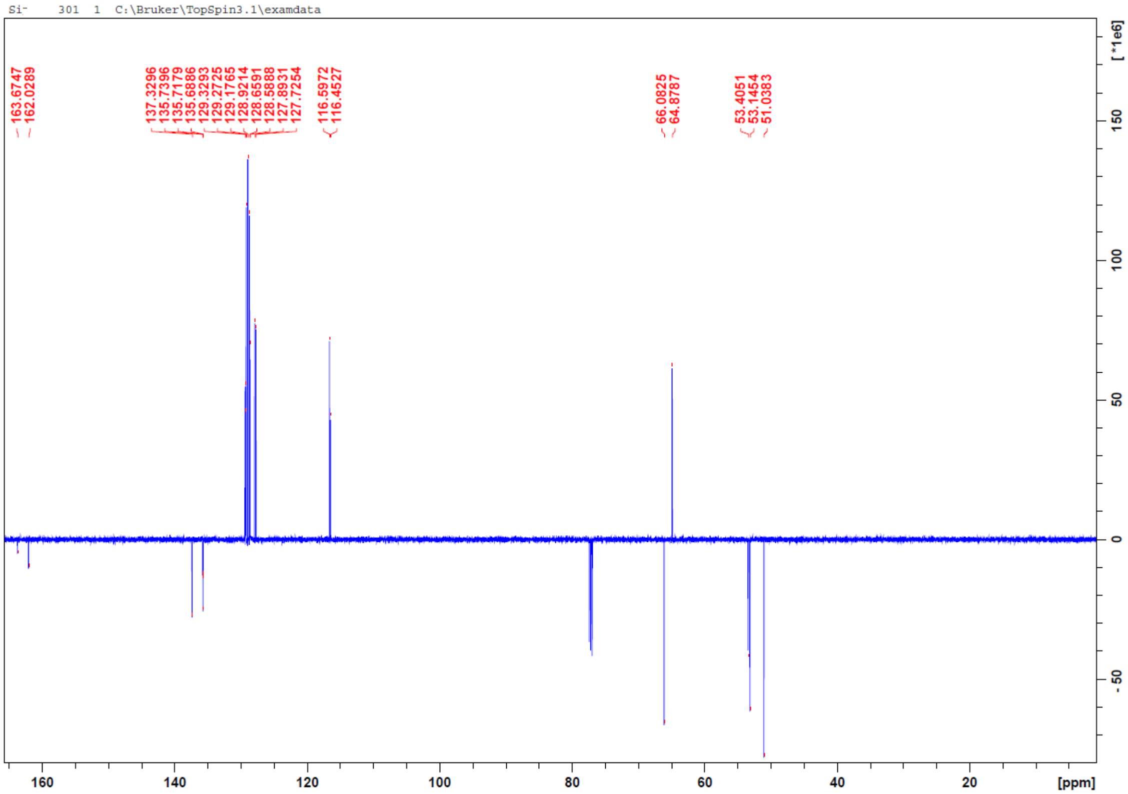

Figure S30. [13] C NMR (100 MHz, $CDCl_3$) of **2,4-dibenzyl-5-(4-fluorophenyl)-1,2,4-thiadiazinane 1,1-dioxide (7h)**

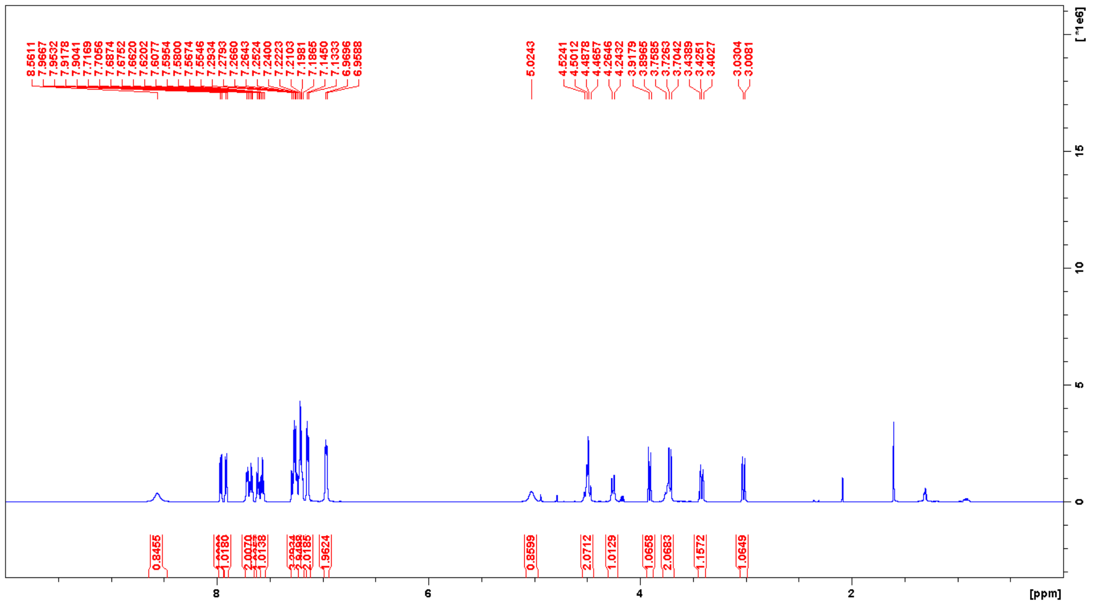

Figure S31. ^{1}H NMR (400 MHz, $CDCl_3$) of **2,4-dibenzyl-5-(4a,8a-dihydronaphthalen-2-yl)-1,2,4-thiadiazinane 1,1-dioxide (7i)**

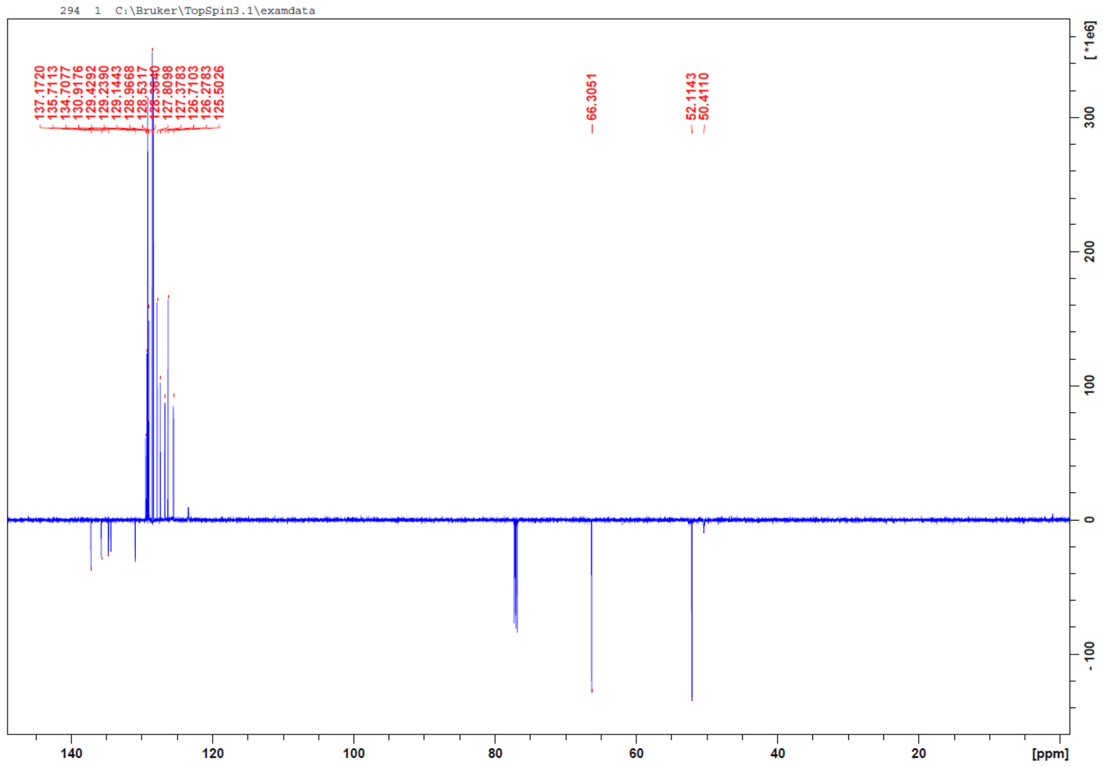

Figure S32. ^{13}C NMR (100 MHz, $CDCl_3$) of **2,4-dibenzyl-5-(4a,8a-dihydronaphthalen-2-yl)-1,2,4-thiadiazinane 1,1-dioxide (7i)**
